# Integration of domestic wastewater and native *Tetradesmus obliquus* for bioremediation and production of biomass rich in protein and polyunsaturated fatty acids

**DOI:** 10.1007/s11356-026-38213-w

**Published:** 2026-09-09

**Authors:** Diego Serrasol do Amaral, Leonardo Werner, Antônio Duarte Pagano, Amanda Fonseca Leitzke, Daisa Hakbart Boneman, Diogo La Rosa Novo, Leandro Silva Nunes, Danielle Tapia Bueno, Luciane Varini Laporta, Rosane Lopes Crizel, Alaor Valério Filho, Vinicius Farias Campos, Claudio Martin Pereira de Pereira

**Affiliations:** 1 Laboratory of Innovation and Solutions in Chemistry, Federal University of Pelotas (UFPel), Capão Do Leão, RS Brazil; 2 Laboratory of Structural Genomics, Federal University of Pelotas (UFPel), Capão Do Leão, RS Brazil; 3 Center of Chemical, Pharmaceutical and Food Sciences, Federal University of Pelotas (UFPel), Capão Do Leão, RS Brazil

**Keywords:** Microalgal, Environmental remediation, Fatty acids, Inoculum density, Biochemical composition, Circular bioeconomy

## Abstract

The large-scale deployment of microalgae-based bioprocesses is often limited by high freshwater and nutrient demands. Domestic wastewater represents a sustainable alternative, enabling simultaneous pollutant removal and biomass production. In this study, a native strain of *Tetradesmus obliquus*, isolated in southern Brazil, was cultivated in raw domestic wastewater (RDW) and primary-treated domestic wastewater (TDW) at three initial inoculum densities (10%, 20%, and 30% v/v) for 14 days. *Tetradesmus obliquus* in TDW inoculated at 10% (v/v) removed 62.46% of ammonium and 98.56% of phosphate, simultaneously exhibiting the highest specific growth rate (0.16 d⁻^1^) and the highest biomass productivity (42.54 mg L⁻^1^ d⁻^1^). Iron and manganese concentrations decreased significantly with a native strain in TDW, indicating effective removal of them under the evaluated conditions. RDW was associated with higher carbohydrate accumulation (32.68%) and pigment production, whereas TDW was associated with higher protein content (45.83%) and a lipid fraction with a high relative proportion of polyunsaturated fatty acids (72.70–78.90%), primarily represented by *α*-linolenic and linoleic acids. The combined assessment of wastewater condition and initial inoculum density revealed distinct effects on the cultivation system. These aspects influence the biochemical composition of biomass, initial inoculum density, nutrient removal, specific growth rate, and biomass productivity. Thus, the present study supports the potential to integrate domestic wastewater treatment with the cultivation of a native *Tetradesmus obliquus* strain and indicates that, under the evaluated conditions, both the wastewater treatment conditions and the initial inoculum density influenced bioremediation performance and the biochemical composition of biomass.

## Introduction

The increasing global population and the rising demand for food and energy have intensified challenges related to domestic wastewater management and climate change (Koop and van Leeuwen 2017). The inadequate disposal of domestic wastewater containing high concentrations of nitrogen, phosphorus, and metals represents a major source of environmental pollution, contributing to eutrophication processes and biodiversity losses in aquatic ecosystems (Wear et al. 2021; Tuholske et al. 2021). Although wastewater treatment plants commonly rely on physicochemical and biological processes, these conventional approaches are often associated with high energy consumption, elevated operational costs, and the generation of secondary sludge (Crini and Lichtfouse 2019). In this context, the development of sustainable strategies aligned with circular bioeconomy principles has become essential to promote nutrient recovery and the biotransformation of effluents into value-added bioproducts (Śniatała et al. 2024; Cheng et al. 2025).

The use of microalgae for domestic wastewater biotransformation has emerged as an innovative and sustainable alternative (Wágner et al. 2021; Mubashar et al. 2024). However, large-scale microalgal production remains economically constrained by high water and nutrient demands, particularly nitrogen and phosphorus, as well as significant downstream processing costs (Borowitzka and Vonshak 2017; Novoveská et al. 2023). Thus, the integration of domestic wastewater as a nutrient source for microalgal cultivation represents a promising circular approach for reducing production costs while enabling sustainable biomass generation (Allen et al. 2018; Do et al. 2019). Consistently, recent reports in the literature indicate that replacing synthetic culture media with wastewater can substantially reduce nutrient and freshwater inputs and biomass production costs, reinforcing the rationale for coupling effluent treatment with biomass generation within a circular bioeconomy (Dias et al. 2025). Nevertheless, microalgal cultivation in domestic wastewater can be challenged by variability in effluent composition and cultivation conditions (Gouveia et al. 2016; Wang et al. 2017). These limitations may be mitigated through the selection of native microalgae strains, which are adapted to local climatic conditions and effluent variability (Rocha et al. 2017; Sisman-Aydin 2022). Indeed, previous studies have demonstrated that native strains can grow rapidly and efficiently remove nutrients from wastewater (Lynch et al. 2015).

The performance of microalgae-based wastewater treatment systems is influenced by various cultivation parameters, including strain selection, initial inoculum density, climatic conditions, and effluent characteristics, among others, that affect biomass productivity and nutrient removal efficiency (Wang et al. 2017; Petrini et al. 2020; Yang et al. 2023). Inoculum density and light intensity jointly affected microalgal growth and biomass production in primary and secondary municipal wastewater, with the effects varying according to effluent turbidity and nutrient availability (Bohutskyi et al. 2016). These findings highlight the importance of evaluating inoculum density in relation to the wastewater matrix. The initial inoculum density can influence culture establishment by affecting light availability per cell, nutrient availability, self-shading, and growth dynamics relative to the effluent native bacteria. Consequently, variations in inoculum density may affect biomass productivity, nutrient uptake kinetics, and the overall efficiency of wastewater bioremediation (Novoveská et al. 2016; Plöhn et al. 2021; Yang et al. 2023). Likewise, wastewater dilution influences nutrient availability and light penetration, thereby affecting microalgal growth and nutrient removal performance (James et al. 2023; Hassan et al. 2024). Growth dynamics and nutrient availability regulate cellular metabolism, and these operational conditions may also influence the biochemical composition of the resulting biomass (El-Sheekh et al. 2023; Singh et al. 2024). Therefore, evaluating the combined effects of cultivation conditions and effluent characteristics is essential for developing efficient microalgae-based wastewater treatment processes within a circular bioeconomy framework (Abdelaziz et al. 2014; Sisman-Aydin 2022).

Microalgae have gained increasing attention beyond wastewater treatment due to their potential for producing high-value-added biomolecules. Microalgal biomass is a rich source of polyunsaturated fatty acids (PUFAs), pigments, carbohydrates, and proteins with diverse biotechnological applications (Levasseur et al. 2020). Polyunsaturated fatty acids are essential long-chain fatty acids involved in cell membrane structure and signaling processes (Khodadadianzaghmari et al. 2024). Polysaccharides, including exopolysaccharides (EPS), exhibit bioactive and biostimulant properties, promoting plant growth, enhancing nitrogen metabolism, and increasing tolerance to abiotic stresses (El Arroussi et al. 2018; Rachidi et al. 2020). In addition, microalgal proteins and amino acids can serve as readily assimilable organic nitrogen sources, supporting plant development and metabolic efficiency (González et al. 2020; Braun and Colla 2023), as well as contributing to animal feed supplementation (Patnaik et al. 2019). Understanding how cultivation conditions influence both biomass production and biomolecule accumulation is therefore crucial for improving the economic viability of microalgae-based systems.

Recent studies in the literature reinforce the efficacy of native and indigenous microalgae in the simultaneous treatment of various effluents and the recovery of value-added biomass. Among indigenous strains, *Chlorella* sp. and *Desmodesmus* sp. cultivated in domestic wastewater removed up to 99% of nitrate, 100% of phosphate, and 92–98% of heavy metals, achieving biomass productivities of 90–94 mg L⁻^1^ d⁻^1^ and generating metabolites suitable for biofuels and bioactive applications (Sreelekshmy and Ummalyma 2026). Indigenous strains of *Tetradesmus obliquus* and *Tetradesmus dimorphus* inoculated at 50% of raw tannery effluent achieved complete ammonia removal and protein-rich biomass (up to 37.20%) suitable as an animal feed supplement (Ranjan et al. 2025). Similarly, a native strain of *Tetradesmus obliquus* in anaerobically digested dairy wastewater achieved ≥ 80% removal of ammonium, nitrate, and phosphate and biomass containing 37% protein and 22.40% lipids (Popovich et al. 2025). Collectively, and as systematized in recent frameworks for integrated algae-based biorefineries (Ansari et al. 2026), these studies confirm that effluent-adapted strains can combine efficient bioremediation with the production of proteins, lipids, and other bioproducts from industrial and agricultural wastewater.

Among the operational parameters governing microalgal bioremediation performance in domestic wastewater, initial inoculum density is one of the few that is low-cost, directly controllable, and easily scalable to real-world applications. In practical applications involving non-sterile effluents, the inoculum-to-substrate ratio may influence interactions between the introduced microalgal strain and the indigenous bacterial community. The structure and carbon- and nitrogen-related metabolic functions of native bacteria can respond to variations in microalgal inoculum concentration (Yang et al. 2023), and the evolution from a pure inoculum to a stable microalgae-bacteria consortium in real wastewater is itself governed by the inoculation strategy (Petrini et al. 2020). A higher inoculum favors rapid microalgal establishment, whereas a lower inoculum leaves more room for the resident microbiota to compete for nutrients during the initial phase. Furthermore, inoculum density determines the internal light environment of the culture through mutual self-shading. As cell concentration increases, the irradiance available to individual cells decreases, an effect that may be further intensified by the turbidity and color of domestic wastewater (Bohutskyi et al. 2016; Martínez et al. 2018). These opposing effects may create a trade-off between final biomass accumulation and specific growth rate, while also influencing nutrient removal through changes in the number of active cells and their physiological state (Lau et al. 1995). Despite this operational significance, the influence of initial inoculum density on the simultaneous objectives of biomass production and nutrient/metal removal by native strains in domestic wastewater remains insufficiently characterized.

Despite these advances, significant knowledge gaps remain, and most studies have focused on industrial or agro-industrial wastewater, whereas comparatively only a few studies have evaluated native freshwater strains of the genus *Tetradesmus* cultivated in domestic wastewater. Furthermore, the combined influence of the effluent treatment level and the initial inoculum density on nutrient removal, biomass productivity, and biochemical composition have been scarcely investigated. This limitation hinders a comprehensive understanding of how operating conditions affect both wastewater bioremediation and biomass valorization within circular bioeconomy systems. The present study addresses these gaps by combining a native *Tetradesmus obliquus* strain from southern Brazil with a paired comparison between raw and primary-treated domestic wastewater, using three initial inoculum densities; this establishes an explicit relationship between operating conditions and both nutrient/metal removal and the production of a PUFA-rich biochemical profile. This experimental design enabled the simultaneous evaluation of: (i) the influence of the effluent treatment level; (ii) the effect of initial inoculum density on biomass production and nutrient removal; and (iii) the biochemical composition of the resulting biomass.

## Materials and methods

### Sampling and microalgae isolation

Domestic wastewater samples were collected in sterile 50 mL polyethylene tubes at the wastewater treatment station of the Federal University of Pelotas—Capão do Leão Campus, Pelotas, Brazil, (− 31.8014533, − 52.4150785), and maintained at room temperature (21 ± 2 °C) until processing. Microalgal isolation was initiated by inoculating 100 µL of the collected wastewater into 150 mL of Basal Bold Medium (BBM) under constant aeration and continuous illumination (70 µmol photons m⁻^2^.s⁻^1^) (Andersen and Kawachi 2005). Following the establishment of the mixed microalgal culture, serial dilutions were performed in 24-well plates to facilitate the isolation of unialgal cultures (Andersen and Kawachi 2005). The resulting cultures were examined by light microscopy (Olympus CX41, Olympus, Japan) to identify wells containing morphologically unialgal cultures. Subsequently, 50 μL of the culture was inoculated onto Bold's Basal Medium (BBM) solidified with 1.5% agar (98%, Dinâmica Química, Brazil). A single isolated colony was then selected and transferred to 5 mL of fresh BBM medium in 15-mL polyethylene tubes to establish the unialgal working culture. Preliminary taxonomic identification of the isolate was performed by the same light microscopy (Olympus CX41) at 400 times of magnification, based on morphological characteristics described in the literature (Andersen and Kawachi 2005; Prandini et al. 2016; Silva et al. 2023).

### Molecular and phylogenetic analyses

Total deoxyribonucleic acid (DNA) from the isolated strain was extracted from 15 mL of liquid culture using the Wizard® Genomic DNA Purification Kit (Promega, USA). Total DNA concentration and purity were assessed using a NanoDrop™ Plus spectrophotometer (Thermo Fisher Scientific, USA). Molecular identification was performed by amplifying fragments of the 18S ribosomal ribonucleic acid (RNA) gene using conventional polymerase chain reaction (PCR) using GoTaq® G2 Flexi DNA Polymerase (Promega Corporation, USA). The primers were 5′-GTCAGAGGTGAAATTCTTGGATTTA-3′ (forward) and 5′- AGGGCAGGGACGTAATCAACG-3′ (reverse) as described by Khaw et al. (2020). The amplification protocol consisted of an initial denaturation at 94 °C for 1 min, followed by 35 cycles of 94 °C for 30 s, annealing at 63 °C for 30 s, and extension at 72 °C for 1 min, with a final extension at 72 °C for 5 min. Amplification products were verified by electrophoresis on a 1.2% agarose gel.

Polymerase chain reaction products were purified using E-Gel™ EX (Invitrogen™, USA) and sequenced by the Sanger method using an Applied Biosystems 3500 Genetic Analyzer® (Life Technologies, USA). Sequence quality assessment and preliminary identification were performed by comparison with sequences deposited in GenBank®. For taxonomic identification of the novel isolated strain, reference sequences were retrieved from GenBank via Basic Local Alignment Search Tool for nucleotides (BLASTn) searches using the sequenced 18S sequence. The nucleotide sequences were aligned using the MAFFT version 7 web server (https://MAFFT.cbrc.jp/alignment/server/index.html). Non-overlapping regions at the sequence ends were trimmed with MEGA11, and reference sequences containing missing data relative to the isolates were excluded. For tree rooting, *Chlamydomonas reinhardtii* strain SAG 53.72 (GenBank Accession Number JN903984.1) was included as an outgroup. Phylogenetic analysis was performed using the IQ-TREE server (http://iqtree.cibiv.univie.ac.at/), employing maximum likelihood phylogenetic inference. The ModelFinder application selected the most appropriate evolutionary model based on the Bayesian information criterion (BIC). Branch support was evaluated through Ultrafast Bootstrap Approximation (UFBoot) with 1000 replicates (Minh et al. 2013), along with an approximate likelihood-ratio test akin to the Shimodaira–Hasegawa approximate likelihood ratio test (SH-aLRT) procedure with 1000 replicates (Anisimova and Gascuel 2006) and an approximate Bayes test.

### Sampling and characterization of domestic wastewater

Domestic wastewater used for microalgae production was collected from the same location described in Section. 2.1. Two types of domestic wastewater were evaluated for the cultivation of *Tetradesmus obliquus*: raw domestic wastewater (RDW) and domestic wastewater subjected to primary-treated domestic wastewater (TDW). Primary treatment consisted of nylon mesh filtration followed by chemical flocculation using 550 mg L⁻^1^ of dissolved aluminum sulfate salt (98%, Vetec Química, Brazil) solution and sedimentation for 1 h (Zhou et al. 2008). The optical density of the effluents was measured at 680 nm (OD_680_) using a UV–Vis spectrophotometer model UV-M51 (BEL Engineering, Italy) with a 1 cm optical path quartz cuvette. Measurements of pH, electrical conductivity (EC), and total dissolved solids (TDS) were performed immediately after sampling using a multiparameter analyzer model AKSO AK88 (AKSO, Brazil), following the manufacturer's calibration procedures (Beltrán-Rocha et al. 2024).

Ammonium and phosphate concentrations were determined colorimetrically, as described in the Standard Methods for the Examination of Water and Wastewater, following filtration through GF/6 glass fiber filters (45 mm diameter) using a UV–Vis spectrophotometer (UV-M51, BEL Engineering) equipped with a 1 cm optical-path quartz cuvette (APHA, 2017). Orthophosphate was determined by the ascorbic acid–molybdate colorimetric method. Phosphate standards were prepared using KH₂PO₄ salt (99%, Dinâmica Química). The color reagent was prepared immediately before analysis by mixing 50 mL of 5 mol L⁻^1^ sulfuric acid solution from concentrated sulfuric acid (98%, Synth, Brazil), 5 mL of 30 g L⁻^1^ dissolved ammonium molybdate salt (83%, Dinâmica Química, Brazil), 15 mL of 54 g L⁻^1^ dissolved ascorbic acid from its salt (99%, Vetec Química), and 5 mL of 1.36 g L⁻^1^ dissolved potassium antimony tartrate salt (99%, Dinâmica Química). For analysis, 0.8 mL of each sample was mixed with 3.2 mL of the freshly prepared color reagent, homogenized, incubated for 30 min at room temperature protected from light, and the absorbance was measured at 880 nm. Ammonium was determined by the phenol–nitroprusside colorimetric method. Ammonium standards were prepared using (NH₄)₂SO₄ salt (99%, Dinâmica Química). The reaction mixture consisted of 0.25 mL of 100 g L⁻^1^ phenol solution prepared from phenol (99%, Dinâmica Química), 0.25 mL of 5 g L⁻^1^ sodium nitroprusside solution prepared from sodium nitroprusside salt (99%, Dinâmica Química), and 0.5 mL of a freshly prepared reagent obtained by mixing equal volumes of 320 g L⁻^1^ sodium citrate solution prepared from sodium citrate (99%, Synth), containing 10 g L⁻^1^ sodium hydroxide (99%, Isofar, Brazil), and 4 g L⁻^1^ dichloroisocyanuric acid solution prepared from dichloroisocyanuric acid salt (98%, Sigma-Aldrich, USA). The reagents were added to 5.0 mL of each sample, homogenized, incubated in the dark for 2 h, and the absorbance was measured at 640 nm.

Iron and manganese concentrations were determined by flame atomic absorption spectrometry (F-AAS), whereas sodium and potassium were determined by flame atomic emission spectrometry (F-AES), after filtration through GF/6 glass fiber filters (45 mm diameter), using an AAnalyst 200 spectrometer (PerkinElmer, Singapore). hollow cathode lamp specific to each analyte was used, together with a deuterium arc lamp for background correction. Sodium and potassium were measured in emission mode. A hollow cathode lamp specific to each analyte was employed, with a deuterium lamp used for background correction (except for Na and K). Acetylene (≥ 99.995%; Linde, Brazil) served as the fuel gas, and compressed air as the oxidant. The burner height was set at 0.5 cm, and the acetylene flow rate was maintained at 2.5 L min⁻^1^ (Bueno et al. 2025). Before analysis, all samples were diluted (1:10) and acidified to pH 2 using concentrated nitric acid (65%, Vetec Química). Calibration curves were prepared using certified metal standard solutions (Dinâmica Química).

### Microalgal biomass production using domestic wastewater

**Fig. 1:**
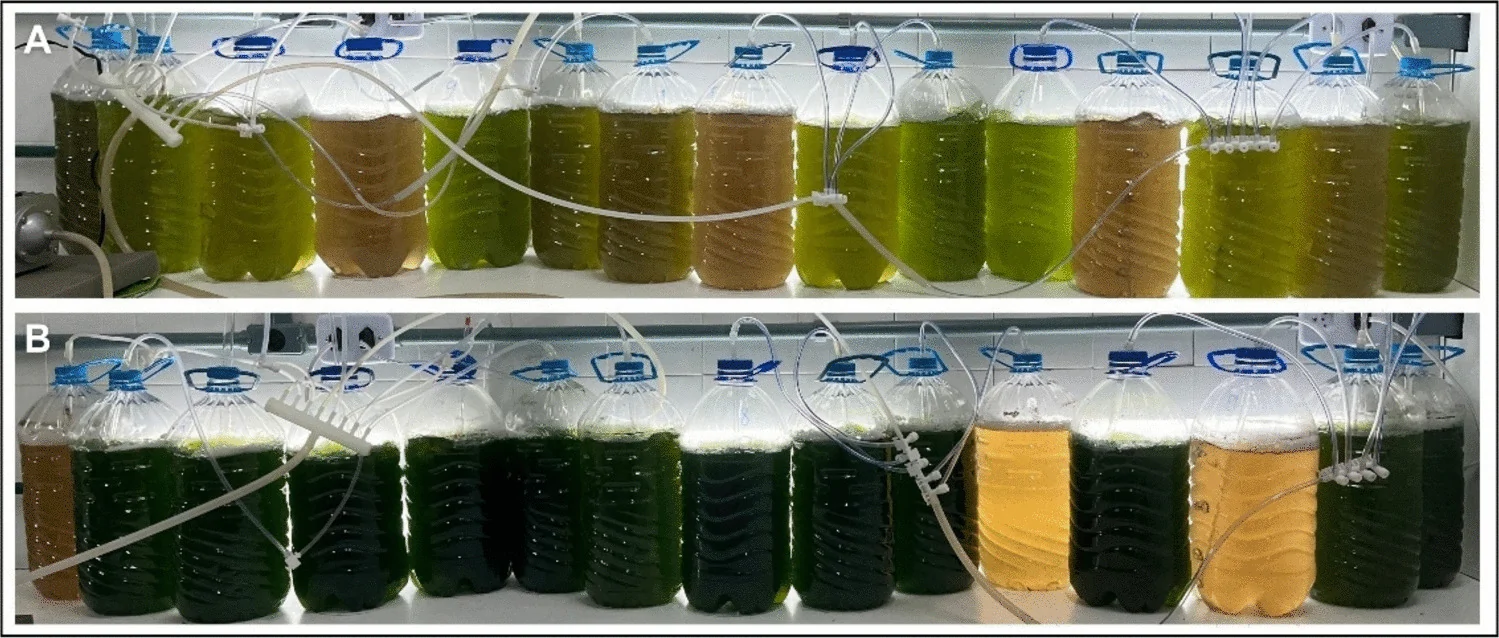
Microalgal biomass production experiment using domestic wastewater. **(A)** Beginning of the experiment (day 0). **(B)** End of the experiment (day 14)

Microalgal cultivation experiments were conducted in 5 L flasks containing 4.5 L of wastewater and different inoculum proportions of *Tetradesmus obliquus* in the exponential growth phase (initial concentration 0.57 ± 0.09 g L⁻^1^) (Fig. 1). For each wastewater, three experimental conditions were established to evaluate the effects of initial biomass concentration and dilution factors on microalgal growth over a 14-day cultivation period. The experimental groups consisted of RDW1 and TDW1 (10% inoculum and 90% wastewater), RDW2 and TDW2 (20% inoculum and 80% wastewater), and RDW3 and TDW3 (30% inoculum and 70% wastewater), resulting in six cultivation conditions. Two additional control groups containing only wastewater (RDW0 and TDW0) were included. All experimental units were maintained under continuous aeration, constant temperature (21 ± 2 °C), and continuous lateral illumination provided by white light-emitting diode (LED) lamps (≈ 150 µmol photons m⁻^2^ s⁻^1^). At the end of the cultivation period, microalgal biomass was allowed to settle for 24 h, followed by centrifugation using a centrifuge model K14-4000 (Kasvi, Brazil) at 4000 rpm for 15 min. The harvested biomass was washed three times with distilled water to remove residual impurities, dried in an oven at 60 °C for 48 h, ground, and stored at − 20 °C until further analyses. Biomass and reagents were weighed on an analytical balance model ATY224R (Shimadzu, Japan).

Samples were collected periodically on days 0, 2, 4, 7, 10, and 14. Dry biomass concentration was determined gravimetrically by filtering 30–50 mL of culture through pre-dried and pre-weighed GF/6 45 mm filters. Optical density was measured directly in homogenized, unfiltered culture samples. The filters were subsequently dried at 60 °C for 24 h, and biomass concentration was calculated from the difference between filter masses before and after filtration. Biomass productivity and specific growth rate (*μ*, day⁻^1^) were calculated based on biomass concentration using Eq. (1), as described by Pandey et al. (2019).

1 $$\mu = \frac{\mathrm{ln}\frac{{X}_{t}}{{X}_{0}}}{{\Delta}_{t}}$$

where ${X}_{t}$ represents the biomass concentration at the end of the experiment (g L^−1^), ${X}_{o}$ is the initial biomass production (g L^−1^), and ${\Delta}_{t}$ is a time variation (day). During the exponential growth phase (approximately days 4–14), μ represented the first-order growth rate constant, whereas biomass productivity represented the average volumetric biomass production over the cultivation period.

#### Physicochemical characterization and evaluation of nutrient and metal removal from effluent during microalgal biomass production

Physicochemical parameters (pH, EC, TDS) and nutrient removal efficiency were evaluated on days 0, 2, 4, 7, 10, and 14. For quantification of ammonium and phosphate, 50 mL aliquots were filtered through glass fiber filters (GF/6, 45 mm) and stored in polyethylene tubes for later analysis, while for the determination of iron, manganese, potassium, and sodium, 50 mL samples were collected in polyethylene tubes at the beginning and end of the experiment (days 0 and 14), both following the same analytical procedures described in Sect. 2.3. The removal efficiency of ammonium, phosphate, iron, manganese, potassium, and sodium was calculated using Eq. (2) and (3) according to the literature (Pandey et al. 2019).

2 $$\mathrm{R}\mathrm{e}\mathrm{m}\mathrm{o}\mathrm{v}\mathrm{a}\mathrm{l}\;\mathrm{e}\mathrm{f}\mathrm{f}\mathrm{i}\mathrm{c}\mathrm{i}\mathrm{e}\mathrm{n}\mathrm{c}\mathrm{y}\; (\mathrm{\%}) = \frac{{C}_{i}-{C}_{f}}{{C}_{i}} X 100$$

3 $$\mathrm{R}\mathrm{e}\mathrm{m}\mathrm{o}\mathrm{v}\mathrm{a}\mathrm{l}\;\mathrm{r}\mathrm{a}\mathrm{t}\mathrm{e}\;(\mathrm{m}\mathrm{g} \mathrm{L}-1 \mathrm{d}-1 ) = \frac{{C}_{f}-{C}_{i}}{{t}_{f}- {t}_{0}}$$

where C_i_ and C_f_ represent the initial and final concentrations of the parameter measured at times t_0_ and t_f_, respectively.

### Biomass characterization analysis

#### Protein

Protein content was determined using the Kjeldahl method (AOAC 2016). Briefly, 200 mg of dried microalgal biomass were digested in a digestion block at 350 °C for 4 h using 10 mL of concentrated sulfuric acid (99%, Synth) and a catalytic mixture of sodium sulfate salt (99%, Dinâmica Química) and copper sulfate salt (99%, Dinâmica Química) at a 7:1 (w/w) ratio. After digestion, the samples were diluted with 50 mL of distilled water, followed by the addition of 40 mL of 50% (w/v) dissolved sodium hydroxide salt (99%, Isofar) solution for distillation. The released ammonia was captured in 30 mL of 4% dissolved boric acid salt (99%, Proquimios, Rio de Janeiro, Brazil) solution containing a mixed indicator of methyl red (95%, Dinâmica Química) and bromocresol green (95%, Dinâmica Química) and, subsequently, titrated with standardized 0,1 mol L^−1^ hydrochloric acid (37%, Synth). A blank was processed under the same conditions. Total protein content was calculated as nitrogen × 6.25 according to the Kjeldahl method (AOAC 2016).

#### Carbohydrates

Total carbohydrates were determined by the phenol–sulfuric acid method (Tan et al. 2024): 500 µL of aqueous solutions of dry biomass (1 mg mL⁻^1^) were reacted with 500 µL of 5% phenol (99%, Dinâmica Química) and 2.5 mL of concentrated sulfuric acid (99%, Synth). The reaction mixture was incubated for 5 min at 90 °C, cooled on ice for 5 min, and the absorbance was measured spectrophotometrically at 490 nm using a UV–Vis spectrophotometer model UV-M51 (BEL Engineering) with a 1 cm optical path quartz cuvette. Glucose (99%, Synth) was used as a calibration standard, and the results were expressed in milligrams of carbohydrate per gram of dry biomass.

#### Pigments

Chlorophyll a and chlorophyll b contents were determined according to the spectrophotometric method described by Jeffrey and Humphrey (1975). Briefly, 25 mg of dried biomass was homogenized in 10 mL of 90% acetone (99.5%, Synth), vortexed for 5 min, and centrifuged at 4,000 g for 10 min. The supernatant was collected, and absorbance was measured at 647, 664, and 750 nm using a UV–Vis spectrophotometer model UV-M51 (BEL Engineering) with a 1 cm optical path quartz cuvette. Chlorophyll concentrations were expressed in mg g⁻^1^ of dry biomass (Jeffrey and Humphrey 1975). Total carotenoids were determined following the method of Rodriguez-Amaya (2001). Dried biomass (50 mg) was extracted with 6 mL of chilled acetone (99.5%, Synth) and vortexed for 3 min. The mixture was transferred to a separatory funnel, followed by the addition of 2 mL of acetone (99.5%, Synth), 3 mL of hexane (99%, Synth), and 3 mL of chilled distilled water. After phase separation, the polar phase was discarded, and the hexane phase was washed three times with 3 mL of distilled water. The final extract was transferred to a 5 mL amber volumetric flask and completed with hexane (99%, Synth). Total carotenoids were then quantified spectrophotometrically at 450 nm using a UV–Vis spectrophotometer model UV-M51 (BEL Engineering) with a 1 cm optical path quartz cuvette, based on the extinction coefficient of *β*-carotene in hexane.

#### Lipid analysis and fatty acid methyl esters profile

Total lipids were extracted using the method described by Bligh and Dyer (1959), and quantified gravimetrically: 200 mg of dried biomass were mixed with 5 mL of chloroform (99.9% Synth), 10 mL of methanol (99% Synth), and 5 mL of 1.5% dissolved sodium sulfate salt (99%, Dinâmica Química) solution and kept under agitation at room temperature for 1 h. Subsequently, 5 mL of chloroform (99% Synth) and 5 mL of 1.5% dissolved sodium sulfate salt (99%, Dinâmica Química) solution were added, followed by an additional 1 min of agitation. After phase separation, the biomass was re-extracted three times with 5 mL of a chloroform (99.9% Synth)–methanol (99% Synth) at a 1:2 ratio and 1 min of agitation for each extraction. All nonpolar phases were pooled, and the solvent was evaporated to dryness. The resulting extract was redissolved in 2 mL of HPLC-grade hexane (≥ 99.99%, Sigma-Aldrich) and evaporated again. The dried extract was weighed and subjected to derivatization with boron trifluoride (99.9%, Sigma-Aldrich) (dos Santos et al. 2021): the extract was refluxed for 5 min with 5 mL of 20 g L⁻^1^ methanolic sodium hydroxide prepared from methanol (99.8%, Synth) and sodium hydroxide (99%, Isofar), followed by the addition of 5 mL of methanolic boron trifluoride (99.9%, Sigma-Aldrich) solution and a second 3 min reflux. Then, 3 mL of saturated dissolved sodium chloride salt (99%, Dinâmica Química) solution and 20 mL of HPLC-grade hexane (≥ 99.99%, Sigma-Aldrich) were added, and the nonpolar phase was separated, dried, and redissolved in HPLC-grade hexane (99.99%, Sigma-Aldrich). Fatty acid composition was expressed as the relative percentage of each identified fatty acid, calculated by chromatographic peak area normalization, without the use of an internal standard.

Fatty acid methyl esters (FAMEs) were analyzed using a gas chromatograph coupled to a mass spectrometer model QP 2020 (Shimadzu, Japan) equipped with an RTX-5MS Supelco capillary column (30 m × 0.25 mm × 0.25 μm) and helium (≥ 99.999%; Linde) as the carrier gas. Chromatographic conditions were 1 μL sample injection under a 1:50 split ratio, helium as the carrier gas at a constant flow rate of 1.2 mL min⁻^1^. The injector was maintained at 250 °C, whereas the interface and ion source temperatures were set to 200 °C. Mass spectra were acquired by electron ionization (70 eV) in full-scan mode over an m/z range of 35–700. The oven temperature was initially set at 100 °C and increased at 3 °C min⁻^1^ to a final temperature of 250 °C, which was held for 5 min, resulting in a total analysis time of 55 min (Berneira et al. 2020). Compound identification was achieved by comparing mass spectra and retention times with the NIST-08 library. Sample preparation is described in Sections. 2.5.4.

#### Ashes

The ash content was quantified gravimetrically following standard analytical protocols (AOAC 2016); 1 g of dry biomass was weighed into previously incinerated porcelain crucibles and incinerated in a muffle furnace at 550 °C for 4 h. After cooling in a desiccator, the ash content was calculated as the percentage of inorganic residue relative to the initial dry biomass mass.

### Statistical analysis

All experiments and analyses were conducted in triplicate, and the results are expressed as mean ± standard deviation. Temporal variations in physicochemical parameters, nutrient and metal removal, and biomass productivity were evaluated using two-way repeated measures ANOVA (RM-ANOVA), followed by Tukey’s post hoc test (*p* < 0.05). Differences among groups in biochemical composition (proteins, lipids, carbohydrates, pigments, and ash) were assessed using a two-way ANOVA, also followed by Tukey’s post hoc test (*p* < 0.05). The Shapiro–Wilk test was applied to verify data normality. All statistical analyses were performed using GraphPad Prism version 9.0 (GraphPad Software, USA).

## Results and discussion

### Microalgal isolation, molecular identification and phylogeny

**Fig. 2:**
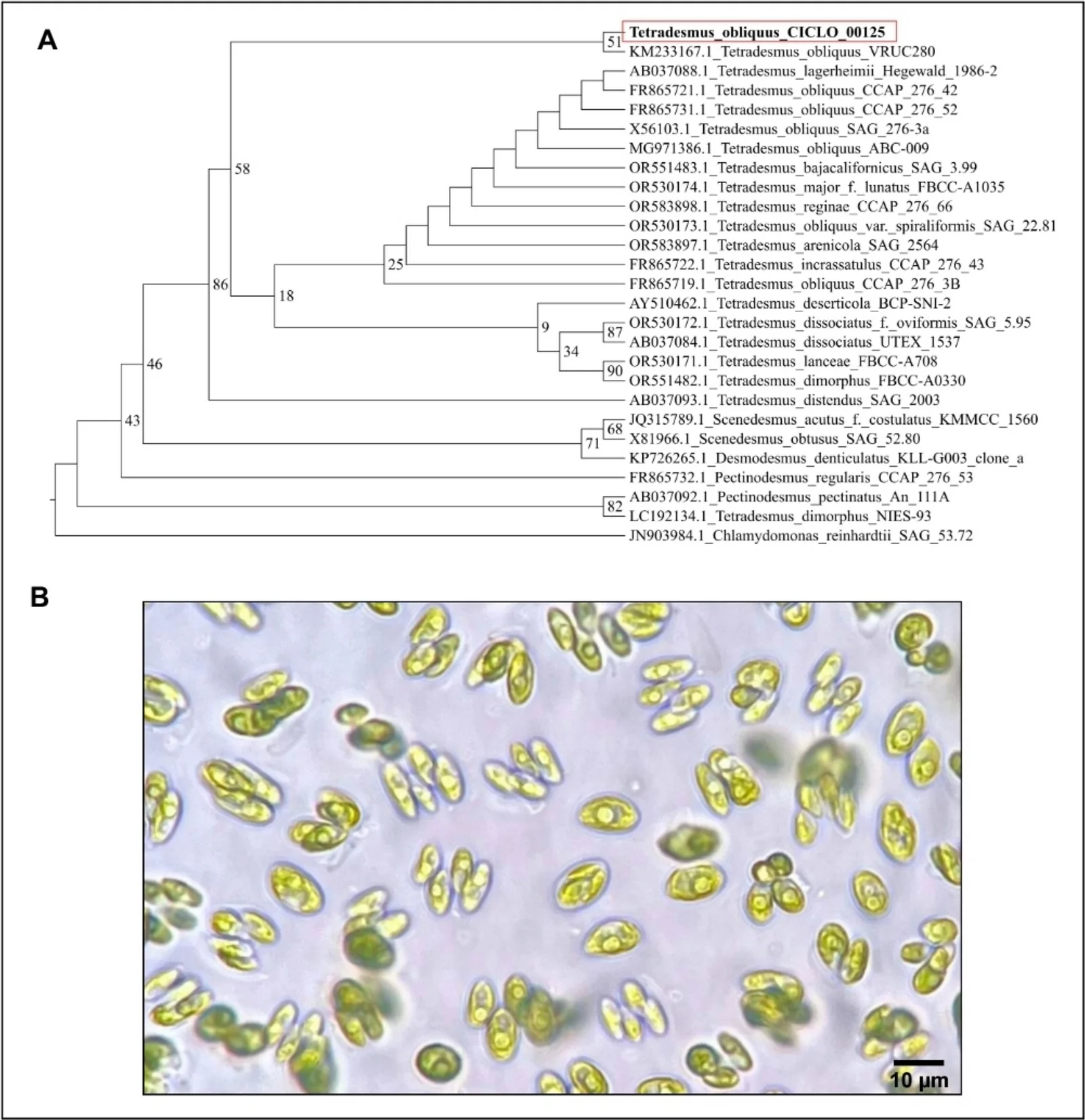
Molecular identification of *Tetradesmus obliquus* strain CICLO_00125*.* (**A**) Phylogenetic tree of the *18 s* gene by FigTree. (**B**) Morphology of the isolated microalga observed using optical microscopy (400 times of magnification)

The native microalgae used in this study were successfully isolated from domestic wastewater collected at a treatment facility located in southern Brazil. Molecular identification was performed by PCR targeting a partial sequence (amplicon size of *651 bp*) from the small subunit ribosomal RNA gene (*18 s*), widely applied in the phylogenetic analysis of green microalgae. The assembled sequence from *Tetradesmus obliquus* strain CICLO_00125 was deposited in the GenBank database under the Accession Number PX631946. Sequencing followed by BLAST alignment revealed 99.69% identity with *Tetradesmus obliquus* reference sequences (Fig. 2a). This result is consistent with previous reports indicating the frequent occurrence of *Tetradesmus* family in eutrophic environments and municipal effluents, attributed to their high tolerance to physicochemical fluctuations and ability to grow in nitrogen- and organic-rich media (Ferro et al. 2018).

Phylogenetic analysis confirmed that the isolated strain clustered within the *Tetradesmus obliquus* clade, forming a well-supported branch (bootstrap > 85%) and validating its taxonomic identity. Furthermore, morphological observations via light microscopy revealed cellular characteristics consistent with descriptions of *Tetradesmusobliquus* (do Carmo Cesário et al. 2021), including coenobia of 4 cells (dimensions: ~ 11–16 µm in length and ~ 3–6 µm in width) and ellipsoidal to ovoid cells occurring singly or in small coenobia (Fig. 2B). *Tetradesmus obliquus* is widely recognized as a promising candidate for the integrated bioremediation of wastewater and biomass production due to its high ammonium and phosphate uptake capacity, rapid growth rate, and metabolic plasticity (Oliveira et al. 2021). In this context, the molecular confirmation of a native *Tetradesmus obliquus* strain isolated directly from domestic wastewater highlights its intrinsic adaptation to complex effluent matrices. This trait is particularly relevant for circular bioeconomy approaches, as the use of native strains can enhance process stability and nutrient recovery efficiency while reducing the need for rigorous operational control.

### Effluent characterization

The physicochemical characteristics of RDW and TDW are summarized in Table 1. Raw domestic wastewater has shown higher concentrations of ammonium, phosphate, iron, and manganese, as well as high optical density, indicating a high organic load and abundance of suspended particulate matter. This composition suggests enhanced availability of nutrients and micronutrients for microalgal growth; however, elevated turbidity may limit light penetration during the initial cultivation stages, potentially affecting photosynthetic performance (Martínez et al. 2018). In addition, the high organic content of RDW can stimulate heterotrophic bacterial activity, thereby influencing microalgae–bacteria interactions and nutrient assimilation dynamics (Petrini et al. 2020). The presence of iron and manganese in RDW is particularly relevant for *Tetradesmus* metabolism, as these elements act as essential cofactors in enzymatic processes associated with nitrogen assimilation and photosynthetic electron transport (Fox and Zimba 2018). In contrast, TDW has shown higher pH, EC, and TDS, reflecting a more ionic composition, with increased concentrations of sodium and potassium (Table 1). These characteristics are consistent with the removal of particulate matter and associated metals during primary treatment. No detectable concentrations of iron and manganese were observed in TDW, which may influence microalgal metabolic performance due to the absence of these micronutrients required for key physiological functions (Fox and Zimba 2018).

**Table 1:** Physicochemical characterization of raw domestic wastewater (RDW) and primary-treated domestic wastewater (TDW) used in the experiments (mean ± standard deviation, *n* = 3)

Parameter	RDW	TDW
pH	7.38 ± 0.05	7.83 ± 0.05
EC** (µS cm^−1^)	1304.33 ± 41.52	1962.30 ± 74.20
TDS*** (mg L^−1^)	891.00 ± 9.53	1403.30 ± 47.25
OD_680****_	0.34 ± 0.01	0.07 ± 0.03
PO₄^3^⁻ (mg L^−1^)	7.22 ± 0.09	4.03 ± 0.38
NH₄⁺ (mg L^−1^)	92.16 ± 0.46	86.08 ± 0.42
Fe (mg L^−1^)	0.69 ± 0.03	nd*
K (mg L^−1^)	43.86 ± 6.27	76.44 ± 4.30
Mn (mg L^−1^)	0.14 ± 0.01	nd*
Na (mg L^−1^)	58.64 ± 2.56	84.73 ± 6.29

*nd = not detected. **Electrical conductivity (EC),*** Total solids dissolved (TDS), ****Optical density at 680 nm (OD_680_)

Primary treatment produced distinct changes in the physicochemical characteristics of wastewater (Table 1). Optical density decreased from 0.34 to 0.07, while iron and manganese concentrations decreased from 0.69 and 0.14 mg L⁻^1^, respectively, to below the detection limit. Phosphate and ammonium concentrations also decreased, from 7.22 to 4.03 mg L⁻^1^ and from 92.16 to 86.08 mg L⁻^1^, respectively. These changes are consistent with the coagulation–flocculation process, in which aluminum sulfate destabilizes suspended and colloidal particles, promoting their incorporation into Al(OH)₃ flocs and subsequent removal by sedimentation (Zhou et al. 2008). The moderate increase in pH from 7.38 to 7.83 may reflect changes in the acid–base equilibrium associated with coagulant addition and the removal of suspended and colloidal material. Electrical conductivity increased from 1304.33 to 1962.30 µS cm⁻^1^, while total dissolved solids increased from 891.00 to 1403.30 mg L⁻^1^. These changes are consistent with the addition of aluminum sulfate during treatment and the resulting modification of the dissolved ionic matrix. Sulfate released from the coagulant remains in the aqueous phase, while the destabilization of colloidal material may also alter the distribution of dissolved and particulate ions (Zhou et al. 2008; Beltrán-Rocha et al. 2024).

In contrast, the increases in potassium and sodium concentration, from 43.86 to 76.44 mg L⁻^1^ and from 58.64 to 84.73 mg L⁻^1^, respectively, cannot be directly attributed to aluminum sulfate, which contains neither potassium nor sodium. These changes may reflect a combination of variability in the wastewater matrix and changes in the partitioning of monovalent ions between dissolved and particulate phases during treatment. However, the relative contribution of these processes cannot be resolved from the present experimental design. Despite the higher electrical conductivity and total dissolved solids measured in TDW, these conditions did not prevent the growth of *Tetradesmus obliquus* or its nutrient-removal performance. Thus, within the physicochemical range evaluated in this study, the primary treatment modified the wastewater matrix without precluding the subsequent cultivation of the native microalgal strain. Although the reduced turbidity of TDW may favor light penetration and photosynthetic activity, the lower availability of nutrients and trace elements highlights a trade-off between optical conditions and nutritional composition. These contrasting physicochemical profiles of RDW and TDW provide distinct cultivation environments, allowing the assessment of how effluent composition influences microalgal growth, nutrient recovery, and biomass quality.

### Bioremediation performance

#### Physicochemical parameters

**Fig. 3:**
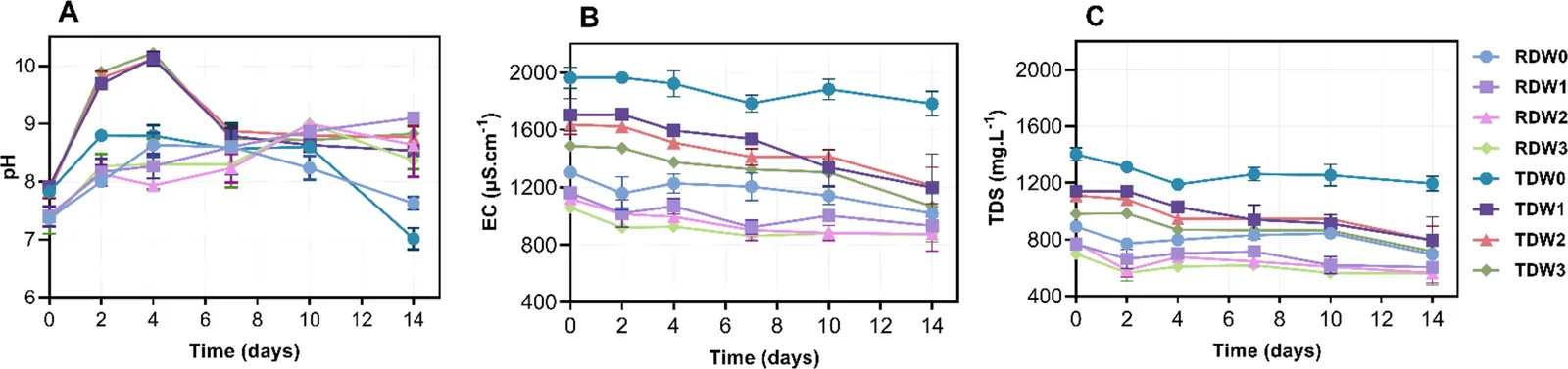
Physicochemical parameters, **(A)** pH; **(B)** Electrical Conductivity (EC); and **(C)** Total Dissolved Solids (TDS) measured during the cultivation of *Tetradesmus obliquus* experiment using three initial inoculum proportions (0: 0%; 1: 10%; 2: 20%; 3: 30%) in domestic wastewater (RDW: raw domestic wastewater; TDW: primary-treated domestic wastewater). Error bars represent standard deviations (*n* = 3)

The evolution of physicochemical parameters, including pH, EC, and TDS, during the 14-day cultivation period is shown in Fig. 3. These parameters varied with wastewater type and initial inoculum proportion. These parameters varied with both wastewater type and initial inoculum proportion. Overall, pH increased more markedly in TDW than in RDW, particularly in TDW2 and TDW3, where values exceeded 10.0 after four days of cultivation (Fig. 3A). This behavior is associated with the higher transparency of TDW, which enhances light penetration and promotes photosynthetic activity (Martínez et al. 2018). During photosynthesis, *Tetradesmus obliquus* consumes dissolved CO₂, shifting the carbonate equilibrium and resulting in hydroxide ion release, thereby progressively increasing medium alkalinity (Plöhn et al. 2021; James et al. 2023). Elevated pH conditions may favor ammonium volatilization and phosphorus precipitation, contributing to nutrient removal; however, these processes can simultaneously reduce nutrient bioavailability for cellular assimilation, potentially constraining biomass accumulation at later cultivation stages (Rawat et al. 2011). In addition, increased alkalinity can suppress heterotrophic bacterial activity, which may reduce microbial competition and favor microalgal predominance (Choi et al. 2010). By day 14, all inoculated treatments exhibited significantly higher pH values than the non-inoculated controls (*p* < 0.001), which remained closer to neutrality (7.62 ± 0.11 for RDW0 and 7.01 ± 0.18 for TDW0), indicating that pH dynamics were primarily driven by microalgal metabolic activity.

Electrical Conductivity (Fig. 3B) and TDS (Fig. 3C) decreased significantly in all inoculated treatments throughout the cultivation period, with inoculum density accounting for a substantial proportion of the observed variance (*p* < 0.0001). The most pronounced reductions in EC and TDS were observed in TDW1, suggesting that lower initial inoculum densities may favor more efficient ion uptake under these conditions. These decreases reflect both the metabolic assimilation of dissolved ions, including ammonium, phosphate, potassium, sodium, iron, and manganese, into cellular components (Xiao et al. 2023; Beltrán-Rocha et al. 2024), and the adsorption of solutes by EPS, whose functional groups facilitate ion binding (Peng et al. 2020). Significant reductions in EC and TDS relative to non-inoculated controls were particularly evident in TDW (*p* < 0.0001). The pronounced decline in EC under these conditions indicates enhanced ion and metal removal, likely promoted by increased photosynthetic activity and reduced microbial competition (Zafar et al. 2022; Yang et al. 2023), consistent with previous reports describing the high ion uptake capacity of *Tetradesmus obliquus*. In contrast, the more moderate reductions observed in RDW0 are likely associated with inherent physicochemical processes and background microbial activity within the wastewater matrix (Drewnowski et al. 2019; Nguyen et al. 2024).

#### Nutrient removal

**Fig. 4:**
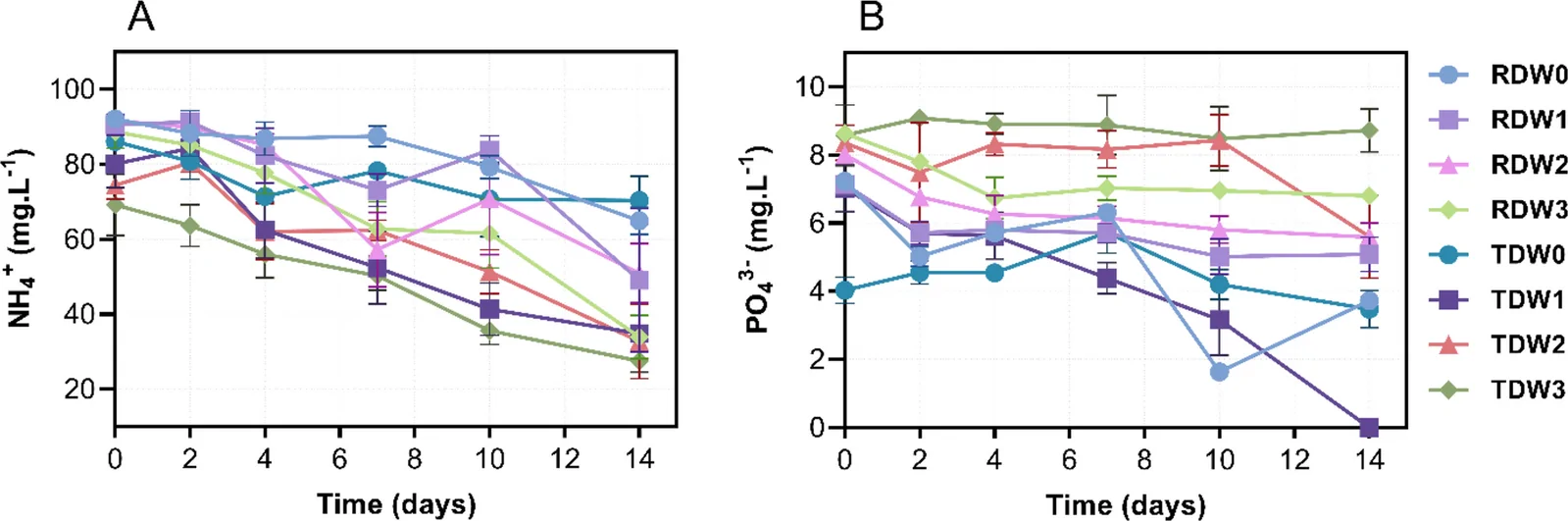
Variation in nutrient concentrations (mg L⁻^1^) during the *Tetradesmus obliquus* cultivation experiment using three initial inoculum proportions (0: 0%; 1: 10%; 2: 20%; 3: 30%) in domestic wastewater (RDW: raw domestic wastewater; TDW: primary-treated domestic wastewater). **(A)** Ammonium (NH₄⁺) concentration during cultivation. **(B)** Orthophosphate (PO₄.^3^⁻) concentration during cultivation. Mean and error bars represent standard deviations (*n* = 3)

Ammonium removal (Fig. 4A) was more consistent in TDW than in RDW. In TDW, the inoculated treatments reached final ammonium concentrations of 27–35 mg L⁻^1^, corresponding to removal efficiencies of 55.68–62.46% and removal rates of 2.97–3.34 mg L⁻^1^ day⁻^1^, whereas the non-inoculated control (TDW0) achieved only 18.24% removal. In RDW, ammonium removal proceeded more slowly, resulting in higher final concentrations (40–63 mg L⁻^1^) and lower efficiencies (30.03–54.07%). Among RDW treatments, RDW3 exhibited the highest ammonium removal rate (3.40 mg L⁻^1^ day⁻^1^, *p* < 0.05), while no significant differences were observed among the remaining groups. Phosphate removal (Fig. 4B) varied markedly among treatments and was strongly influenced by both wastewater type and initial inoculum density. In TDW1, phosphate concentrations declined at 0.49 mg L⁻^1^ day⁻^1^, reaching 98.56% removal by day 14. In contrast, TDW2 and TDW3 achieved only 42.80% and 7.59% phosphate removal, respectively. In RDW, phosphate removal was slower, with final concentrations above 3.70 mg L⁻^1^, efficiencies of 21.17–31.05%, and rates of 0.13–0.15 mg L⁻^1^ day⁻^1^. The RDW control (RDW0) reached 48.36% phosphate removal, indicating the contribution of non-algal processes.

Nitrogen removal in microalgae-based systems reflects a combination of biological assimilation, microorganism-mediated transformations, and abiotic losses, with relative contributions varying according to effluent composition and pH. Photoautotrophic assimilation is the pathway most directly associated with biomass valorization: ammonium, the preferred and energetically least costly nitrogen source, is incorporated into amino acids via the glutamine synthetase-glutamate synthase (GS-GOGAT) cycle (Sanz-Luque et al. 2015). This is consistent with the reduction in ammonium levels observed in the inoculated treatments (TDW: 55.68–62.46%; RDW: 30.03–54.07%) and the low removal observed in the non-inoculated controls (TDW0: 18.24%). A second contribution, abiotic in nature, is ammonia volatilization, which is favored by the shift of the ammonium-ammonia equilibrium toward the free form at high pH; thus, this phenomenon may have been significant in treatments TDW2 and TDW3, where pH values ​​exceeded 10 after the fourth day (Fig. 3A), whereas significant volatilization would not occur in controls with near-neutral pH (RDW0: 7.62; TDW0: 7.01) (Rawat et al. 2011).

Bacterially mediated processes are expected to contribute substantially to nitrogen removal, particularly in RDW. In microalgal–bacterial systems, the two groups are metabolically coupled: microalgae release O₂ that nitrifying bacteria use to oxidize ammonium, while heterotrophic bacteria consume O₂ to oxidize organic matter to inorganic forms and release CO₂ that supports algal photosynthesis (Petrini et al. 2020; Do et al. 2022). In real municipal wastewater, 99% ammonium removal, similar in inoculated and non-inoculated reactors, mainly associated with bacterial activity, has been reported (Petrini et al. 2020). Nitrifying bacteria can dominate ammonium removal when an active indigenous community is present. Heterotrophic and nitrifying populations are favored by high organic loads (Do et al. 2022). Their contribution is expected to be greater in the turbid organically richer RDW (OD₆₈₀ 0.34) than in the clarified TDW, consistent with the highest volumetric ammonium removal rate of this study being recorded in RDW3 (3.40 mg L⁻^1^ d⁻^1^) under conditions least favorable to algal photosynthesis. The structure and carbon/nitrogen metabolic function of the indigenous bacterial community also respond to the microalgal inoculum concentration (Yang et al. 2023), which helps explain the density dependence of nutrient removal observed here. Under alkaline conditions, high pH may additionally inhibit nitrifiers and modulate coupled nitrification–denitrification (Choi et al. 2010; González-Camejo et al. 2018). These bacterial contributions are presented in the present study as plausible and, in a non-axenic system, likely, but not quantified, since the microbial community and process-specific nitrogen fluxes were not measured; the intrinsic risk of competition and contamination by indigenous microorganisms in non-sterile cultivation further supports the need to characterize and manage the microbial community in continuous operation (Bostanci and Koca Akkaya 2025).

Phosphate removal did not scale with biomass production across the TDW treatments, indicating that growth alone does not account for the observed pattern. Although TDW3 reached a final biomass concentration comparable to that of TDW1 (0.68 and 0.66 g L⁻^1^, respectively), its phosphate removal was markedly lower (7.59% versus 98.56%). This decoupling is consistent with the understanding that phosphate removal in microalgal systems is governed largely by the cellular capacity for phosphate uptake and intracellular polyphosphate storage (luxury uptake) rather than by the amount of biomass produced (Powell et al. 2008, 2009). The magnitude of luxury and overplus uptake, which corresponds to the accumulation of phosphorus as polyphosphate under specific physiological transitions, depends on light intensity, external phosphate concentration, temperature, and the physiological state of the cells (Powell et al. 2008; Bossa et al. 2024; Plouviez et al. 2025). Distinguishing these processes would require targeted polyphosphate analyses that were beyond the scope of the present study.

On this basis, the present study hypothesizes that the lower phosphate removal at higher inoculum densities (TDW2 and TDW3) reflects a reduction in the light available per cell caused by mutual self-shading, rather than a decrease in the overall photosynthetic activity of the culture. Self-shading lowers the average irradiance experienced by individual cells, and light intensity has been shown to directly modulate polyphosphate accumulation and luxury phosphorus uptake in microalgae (Powell et al. 2008, 2009). A reduced energy supply at the single-cell level would constrain the ATP-dependent active transport of phosphate and the enzymatic synthesis of polyphosphate (Bossa et al. 2024), even where bulk photosynthetic activity remained high. Consistent with this interpretation, the medium pH in TDW2 and TDW3 exceeded 10 after the fourth day of cultivation (Fig. 3A), confirming that photosynthetic carbon assimilation at the culture level was not suppressed; the limitation therefore appears to operate at the level of per-cell irradiance and phosphorus-uptake physiology. The higher initial biomass in these treatments is also likely to have shortened the exponential phase and advanced the onset of the stationary phase, further reducing the physiological demand for phosphate (Powell et al. 2009; Plouviez et al. 2025).

Phosphate precipitation is thermodynamically controlled by the medium's pH and the availability of precipitating cations, with metal phosphate phases becoming increasingly favored as pH rises (Mulkerrins et al. 2004; Xia et al. 2021). At the pH levels recorded in the TDW treatments (above 10 in TDW2 and TDW3), the precipitation of calcium and magnesium phosphates was expected; however, the lowest phosphate removal occurred precisely in the treatments with the highest pH, contradicting the trend expected in a precipitation-controlled process. This indicates that biological assimilation, rather than abiotic precipitation, was the primary factor determining the differences among the TDW treatments. Furthermore, cation concentration is a limiting parameter for phosphate precipitation (Mulkerrins et al. 2004). The partial phosphate removal observed in the uninoculated raw wastewater control (RDW0, 48.36%) is attributed to non-algal processes occurring within the raw effluent. Biological phosphate removal in wastewater is favored by high organic loading and the availability of readily biodegradable carbon, which support the activity of phosphate-accumulating microbial communities (Mulkerrins et al. 2004). Raw wastewater may combine these conditions (high organic load and active indigenous microbiota) with an abundance of suspended solids (OD₆₈₀ = 0.34, versus 0.07 in TDW) and measurable concentrations of iron and manganese (0.69 and 0.14 mg L⁻^1^, respectively), levels that were below the detection limit in the TDW (Table 1). Under these conditions, phosphate removal in RDW0 likely resulted from the combined action of assimilation by heterotrophic and phosphate-accumulating bacteria, adsorption onto suspended solids, and adsorption or co-precipitation with iron and manganese (oxy)hydroxides; the near-neutral pH of the control makes alkaline precipitation of calcium and magnesium unlikely in this case (Mulkerrins et al. 2004; Xia et al. 2021). In contrast, the TDW0 treatment, which contained neither suspended solids nor detectable iron and manganese, exhibited limited removal, consistent with the reduced abiotic and microbial phosphate-binding capacity of the effluent following primary treatment.

In the RDW cultures, nutrient removal likely reflected the combined contribution of microalgal and indigenous microbial processes. The higher turbidity of RDW (OD₆₈₀ = 0.34) may have reduced light penetration and constrained photosynthetic activity, particularly during the initial cultivation phase, whereas the presence of a native microbial community may have contributed to nutrient transformation and assimilation. Similar contributions of bacterial communities to nutrient removal have been reported in microalgae-based systems treating real domestic wastewater, including substantial ammonium removal in non-inoculated controls (Lau et al. 1995; González-Camejo et al. 2018; Petrini et al. 2020). Accordingly, the lower nutrient removal observed at the lower inoculum densities in RDW (RDW1 and RDW2) may reflect the combined effects of reduced algal biomass and limited light availability, although the relative contributions of microalgae and bacteria were not directly quantified in the present study. The higher nutrient removal observed in the TDW treatments than in RDW is consistent with the lower particulate load and greater light availability than in RDW. These conditions likely favored microalgal growth and nutrient assimilation, as indicated by the higher specific growth rates and the rapid increase in culture pH. The latter may also have contributed to phosphate removal through pH-dependent physicochemical processes, including the precipitation of phosphate-containing minerals (Powell et al. 2009; Popa et al. 2025). Thus, the differences between RDW and TDW likely resulted from the interaction between wastewater characteristics, microbial activity, microalgal growth, and physicochemical processes rather than from a single removal pathway.

In non-axenic microalgal cultures, bacterial processes may also contribute substantially to nitrogen removal. Microalgae–bacteria interactions are metabolically coupled through the exchange of oxygen, carbon dioxide, and organic compounds, and their relative contribution depends on the composition of the wastewater and the prevailing cultivation conditions (Solimeno and García 2017; Petrini et al. 2020; Do et al. 2022). In domestic wastewater, bacterial communities may contribute to ammonium transformation, particularly in systems with an established indigenous microbiota. This possibility is consistent with the high volumetric ammonium removal rate observed in RDW3 (3.40 mg L⁻^1^ d⁻^1^), despite the lower apparent suitability of RDW for photosynthetic activity compared with TDW. However, because bacterial abundance, nitrification rates, and nitrogen transformation pathways were not directly measured, the specific contribution of bacterial processes cannot be quantified from the present dataset.

The strong alkaline conditions reached in some TDW treatments may have further altered the relative contribution of microbial and physicochemical pathways. In TDW2 and TDW3, pH values exceeded 10 after the fourth day (Fig. 3A), conditions that can affect bacterial activity and alter the equilibrium of dissolved inorganic carbon and ammonia species (Choi et al. 2010; Solimeno and García 2017). Under these conditions, the observed nitrogen removal may have resulted from a combination of microalgal assimilation and other physicochemical processes, although the relative contribution of each pathway remains unresolved. Conversely, the lower pH and higher particulate and organic load of RDW may have supported a different balance between algal and bacterial processes. The dependence of nutrient removal on inoculum density may therefore reflect changes in the relative abundance and activity of microalgal and bacterial populations, as previously suggested for microalgae–bacteria systems (Yang et al. 2023).

The initial inorganic nitrogen:phosphorus ratios provide additional context for the observed nutrient-removal patterns. Based on the measured initial ammonium and phosphate concentrations, the ratios were approximately 30 in RDW and 51 in TDW, indicating a marked relative excess of inorganic nitrogen compared with phosphate. Such nutrient imbalance may influence the relative availability of nitrogen and phosphorus during microalgal cultivation, as the balance between these nutrients is an important factor governing microalgal growth and nutrient assimilation in wastewater-based systems (Wágner et al. 2021). In the present study, the high initial nitrogen:phosphorus ratios may have contributed to the more extensive depletion of phosphate and the persistence of residual ammonium observed in the cultures. However, these ratios should be interpreted as approximate, since they were calculated from the measured initial ammonium and phosphate concentrations and do not account for other nitrogen and phosphorus forms or for changes in nutrient availability during cultivation. Phosphate removal was additionally influenced by physicochemical processes associated with the culture conditions, as discussed above.

*Tetradesmus obliquus* is recognized for its high nutrient recovery efficiency and biomass productivity, supporting its application in wastewater treatment and bioproduct generation (Oliveira et al. 2021). Maximum phosphorus and ammonium removal rates of 2.90 ± 0.80 mg L⁻^1^ day⁻^1^ and 28.60 ± 0.90 mg L⁻^1^ day⁻^1^ (with removal efficiencies of 91.70% and 59.70%, respectively) have been reported when *Tetradesmus obliquus* was cultivated in diluted anaerobic effluent (Tan et al. 2024), consistent with the phosphate removal trends observed in this study. Similarly, nitrogen and phosphorus have been removed with efficiencies of 71–85% and 78–92% in 75% diluted effluents (Silambarasan et al. 2023). In the present study, higher initial inoculum densities (RDW3 and TDW3) were associated with greater ammonium removal and higher residual phosphate concentrations, whereas lower inoculum densities (RDW1 and TDW1) resulted in a more balanced removal of these nutrients. This pattern may indicate that nutrient removal was influenced by the interaction between initial inoculum density and effluent composition, although the relative contribution of each factor was not experimentally isolated. Compared with the studies summarized in Table 2, the native *Tetradesmus obliquus* strain evaluated here demonstrated efficient nutrient and trace-metal removal in non-sterile domestic wastewater while producing biomass with high protein and C18-PUFA contents, without extensive dilution or sterilization commonly used in other studies.

**Table 2:** Comparison of recent studies (2016–2026) on microalgal cultivation in wastewater for nutrient/pollutant removal and biomass production

Microalgae	Wastewater type	Nutrient/pollutant removal	Biomass/productivity	Key outcome	Reference
*Chlorella* and *Scenedesmus* (mono- and poly-culture)	Primary and secondary municipal wastewater plus anaerobic effluent	Both effluents provided cost-effective nutrient sources for microalgal cultivation and sustainable biofuel production	40–60 mg L^−1^ d^−1^	Higher inoculum densities increased final biomass concentration (up to 380 mg L^−1^), whereas inocula above 10^5^ cells mL^−1^ did not substantially improve lipid content or composition	Bohutskyi et al. (2016)
Native *Nostoc muscorum*, *Navicula veneta*, *Chlorella vulgaris*	Primary-treated municipal wastewater	COD* 85.7–95.7%; TN** 72.0–96.9%; TP*** 86.07–99.8%	*N. veneta* achieved a 0.88 ± 0.04 d^−1^ growth rate and 50.45 ± 1.5 mg L^−1^ chlorophyll-a biomass yield	Native microalgae effectively reduced TN, TP, and COD	Sisman-Aydin (2022)
*Scenedesmus* sp.	Diluted municipal wastewater (75%)	COD* 91.4%; NH_4_^+^ 88.4%; TN** 93.3%; TP*** 96.3%	1.93 g L^−1^ biomass; protein 35%, carbohydrate 20.4%, lipid 33%	*Scenedesmus* sp. enabled wastewater treatment while producing lipid-rich biomass and 378 mg L^−1^ exopolysaccharides with biostimulant potential	Silambarasan et al. (2023)
*Tetradesmus obliquus*	Raw domestic wastewater and recycled algal effluent	NO_3_^−^ > 70%, NH_4_^+^ > 84%, PO_4_^3−^ > 87.57%, and COD* up to 87.78% removal across wastewater-based media	1.59 g L^−1^ biomass (75% raw wastewater and 25% recycled effluent); lipids 32.16%, protein 33.32%	A recycled-effluent/raw-wastewater mixture optimized biomass production, CO_2_ fixation (0.19 g CO_2_ L^−1^ d^−1^), and resource recovery while reducing freshwater and nutrient demand	Hassan et al. (2024)
*Scenedesmus obliquus*	Anaerobically digested swine wastewater (different cleaning processes)	Removed up to 94.2% COD*, 91.7% NH_4_^+^, and 83.2% P	Biomass 1.46–2.47 g L^−1^	Lipid production reached 12.1–19.4 mg L^−1^ d^−1^, with saturated fatty acids comprising 27.9–36.4% of total fatty acids, supporting biodiesel applications	Tan et al. (2024)
*Chlorella vulgaris*	Unsterilised tertiary effluent and anaerobic-digestion centrate (60 L raceway, 90 d)	NH_4_^+^ removal reached approximately 30 mg L^−1^ d^−1^	Suspended solids up to 770 mg L^−1^ in the raceway reactor	Recirculated sedimentation-controlled contamination and enabled 90 days of operation in an unsterilized wastewater-based microalgal culture	Bostanci and Koca Akkaya (2025)
Native *Tetradesmus obliquus*	Dairy-farm wastewater digestate (post-anaerobic digestion)	NH_4_^+^, NO_3_^−^, and PO_4_^3−^ removal ≥ 80%	104 mg L^−1^ d^−1^ (vs. 74 in BBM****); protein 37% dry weight; lipid 22.4% dry weight; phytosterol-rich	A native isolated strain efficiently phycoremediated post-anaerobic-digestion dairy effluent, adapting to a turbid, high-N:P medium and coupling treatment with resource recovery	Popovich et al. (2025)
Indigenous *Tetradesmus obliquus*, *Tetradesmus dimorphus*, and *Neochloris* sp.	Raw tannery effluent (50%)	NH_3_ removal 12 mg L^−1^ (99%)	Biomass 1.11–1.50 g L^−1^; carbohydrate 12.7–19.8%, protein 24.5–37.2%, fatty acids 7.4–13.3% w/w	Indigenous strains acclimated to raw tannery effluent; their large coenobia settled spontaneously, enabling low-cost harvesting without chemical or physical intervention, supporting a tannery biorefinery	Ranjan et al. (2025)
Indigenous *Chlorella* sp. and *Desmodesmus* sp.	Domestic wastewater	NO_3_^−^ 99%; PO_4_^3−^ 100%; heavy metals 92–98%	90.93–93.75 mg L^−1^ d^−1^	Indigenous microalgae simultaneously enabled wastewater remediation and biomass production for potential bioenergy and bioproduct applications	Sreelekshmy and Ummalyma (2026)
Native *Tetradesmus obliquus*	Raw and primary-treated domestic wastewater	NH_4_^+^ up to 62.5%; PO_4_^3−^ up to 98.6% in primary-treated domestic wastewater1 and Fe and Mn below detection in primary-treated domestic wastewater (99%)	0.55–0.78 g L^−1^; up to 42.5 mg L^−1^ d^−1^; protein 36–46%; relative PUFA***** up to 78.9% (chiefly C18)	A native strain achieved bioremediation of non-sterile domestic wastewater, with treatment performance varying according to effluent type and inoculum density	This study

*COD, chemical oxygen demand; **TN, total nitrogen; ***TP, total phosphorus; ****BBM, Bold’s Basal Medium; *****PUFA, polyunsaturated fatty acids; w/w weight per weight. All values are taken from the cited sources; fatty-acid proportions are relative (percentage of total). Bold row: present study

#### Metal removal

**Fig. 5:**
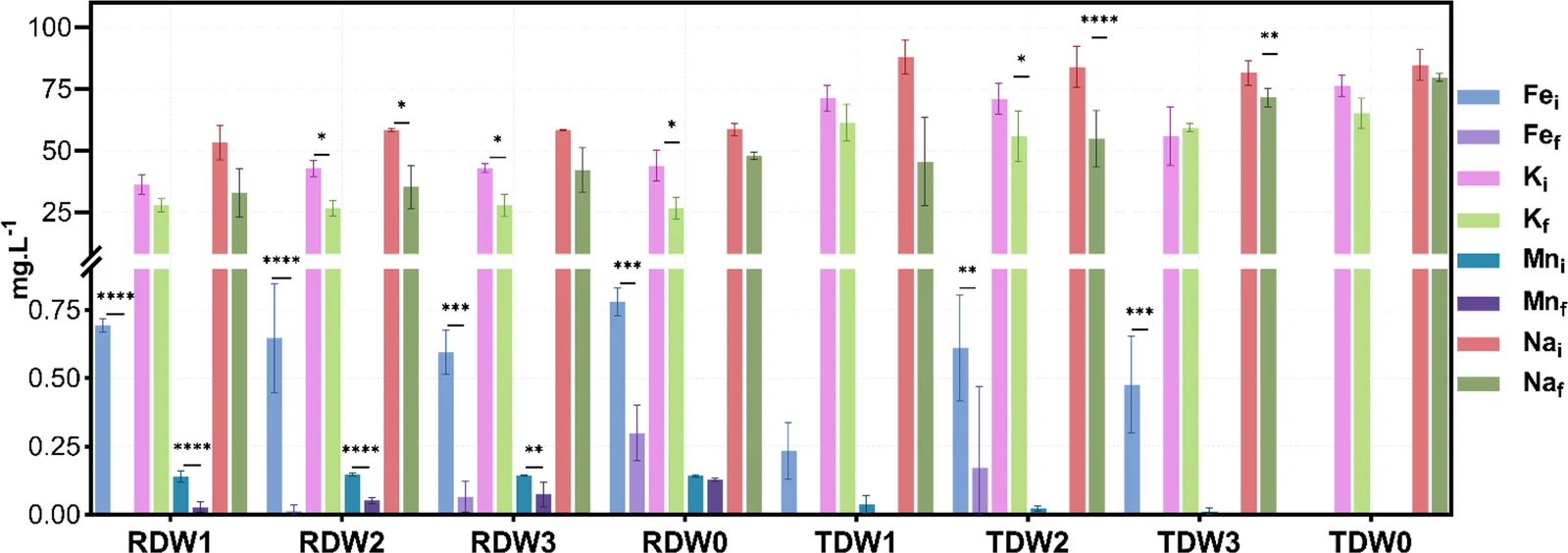
Variation in metal concentrations at the beginning (X_i_) and end (X_f_) of *Tetradesmus obliquus* cultivation in three initial inoculum proportions (1: 10%; 2: 20%; 3: 30%) in different types of domestic wastewater (RDW: raw domestic wastewater; TDW: primary-treated domestic wastewater)

Iron concentrations decreased significantly in both RDW and TDW (*p* < 0.001), with removal strongly dependent on inoculum proportion (p = 0.0019) (Fig. 5). Removal efficiencies reached up to 99% in RDW1 and TDW3, resulting in final iron concentrations below 0.06 mg L⁻^1^ in RDW treatments and 0.17 mg L⁻^1^ in TDW treatments. Manganese removal also showed a significant dependence on inoculum density (p = 0.0021), with the highest removal observed in RDW1, leading to lower final manganese concentrations compared to RDW0. In TDW, both iron and manganese concentrations decreased to below detection limits, with manganese removal efficiencies (80–100%) exceeding the 67.58% previously reported in the literature (Wang et al. 2022). The higher residual iron and manganese concentrations observed in RDW0 highlight the contribution of microalgal inoculation to metal removal. Potassium removal was significant in RDW2, RDW3, and RDW0 (*p* < 0.0001), with the greatest reduction observed in RDW3. In TDW, the most pronounced decrease occurred in TDW2. Sodium removal was highest in RDW1 (*p* < 0.0001) and TDW1, whereas non-inoculated controls exhibited only minor reductions. These trends are consistent with the literature reporting proportional iron uptake and removal efficiencies close to 99% at initial concentrations of 0.2 mg L⁻^1^, particularly in microalgal biofilm systems (Yousefi et al. 2023).

The metal removal performance of the native *Tetradesmus obliquus* strain was primarily governed by wastewater type and, for iron and manganese, by initial inoculum density. These results are consistent with established mechanisms of metal uptake by microalgae, which involve an initial passive adsorption of metal ions onto functional groups present on the cell surface, followed by active intracellular uptake (Subramaniyam et al. 2016; Mahlangu et al. 2024). Iron and manganese removal was attributed to microalgal activity primarily based on comparison with non-inoculated controls, since inoculated treatments consistently reached lower residual concentrations than the corresponding controls, falling below detection in TDW. The absence of a proportional relationship with inoculum density is consistent with the low influent metal concentrations (iron: 0.69 mg L⁻^1^ and manganese: 0.14 mg L⁻^1^, both in RDW): metal removal is dominated by a rapid, saturable passive biosorption onto cell-surface and exopolysaccharides (EPS) functional groups (carboxyl, hydroxyl, and phosphate moieties), which is largely complete even at the lowest biomass, followed by slower active intracellular uptake (Subramaniyam et al. 2016). As iron and manganese act as essential enzymatic cofactors in photosynthesis and respiration (Wang et al. 2022), their availability may further support metabolic activity and indirectly enhance nutrient assimilation processes, including nitrogen and phosphorus removal (Yousefi et al. 2023). Once the available metal pool is nearly depleted, additional biomass does not increase removal proportionally, resulting in a threshold rather than a linear response. Abiotic mechanisms also contributed, including oxidation and co-precipitation of iron and manganese (oxy)hydroxides at the elevated pH and adsorption onto residual coagulant flocs and particulate matter (Subramaniyam et al. 2016). Overall, metal removal reflects the combined action of biosorption, bioaccumulation, and abiotic precipitation, with the net microalgal contribution evidenced by the control comparison. Overall, these results demonstrate the strong bioremediation potential of the native *Tetradesmus obliquus* strain isolated in this study, which efficiently reduced metal concentrations in both RDW and TDW across different inoculum densities. Its adaptation to the physicochemical variability of domestic wastewater and local environmental conditions represents a relevant advantage for practical applications, potentially reducing the need for wastewater dilution, prolonged acclimation phases, and intensive operational control, thereby supporting its integration into circular wastewater treatment and biomass production systems.

### Biomass productivity in different domestic wastewaters

**Fig. 6:**
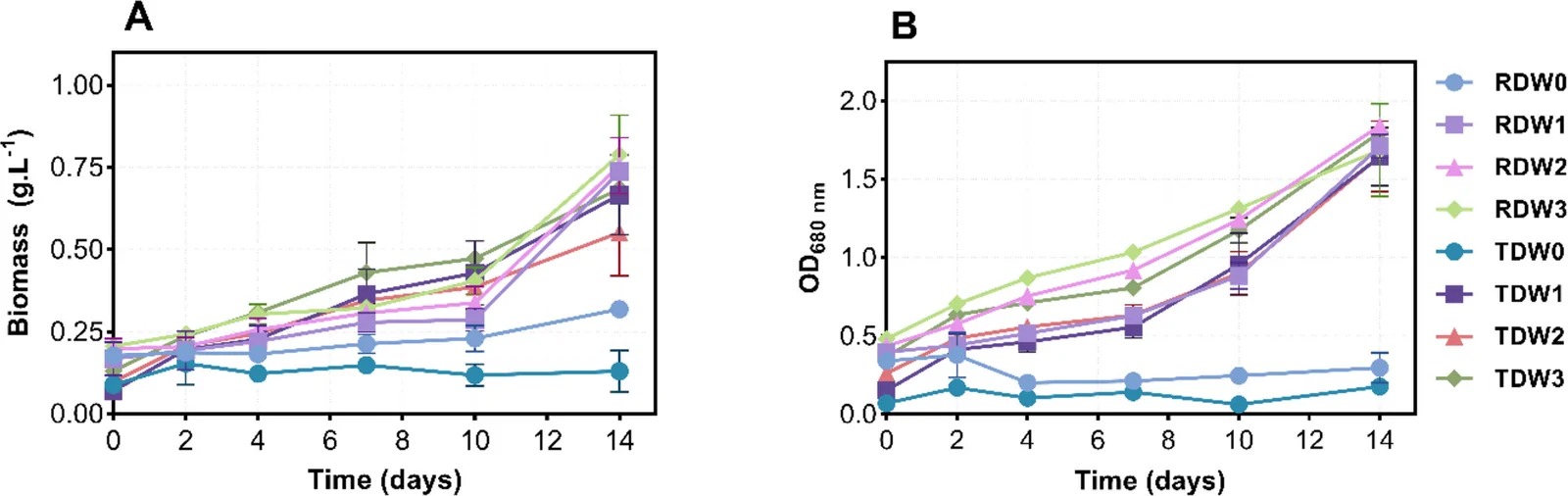
Growth curve of *Tetradesmus obliquus* cultivated at three initial inoculum densities (1: 10%; 2: 20%; 3: 30%) in different types of domestic wastewater (RDW: raw domestic wastewater; TDW: primary-treated domestic wastewater). (A) Dry biomass concentration in RDW and TDW over 14 days. (B) Optical density is at 680 nm during cultivation in RDW and in TDW

Biomass productivity of *Tetradesmus obliquus* cultivated in RDW and TDW was monitored over a 14-day period based on dry biomass concentration and optical density at 680 nm (OD₆₈₀) (Fig. 6). In all treatments, a short adaptation phase was observed during the first four days, followed by exponential growth until the end of the cultivation period. Biomass production differed significantly among treatments, with initial inoculum density accounting for most of the observed variability (*p* < 0.0001). In RDW, final biomass concentration increased progressively with inoculum density, reaching 0.78 ± 0.10 g L⁻^1^ in RDW3, compared with 0.73 ± 0.02 g L⁻^1^ in RDW1 and 0.75 ± 0.08 g L⁻^1^ in RDW2. Despite these differences in final biomass accumulation, biomass productivity remained relatively stable across RDW treatments, ranging from 39.79 ± 8.30 to 41.57 ± 7.80 mg L⁻^1^ d⁻^1^, while the specific growth rate (μ) varied only slightly (0.09 ± 0.01–0.10 ± 0.02 d⁻^1^). In contrast, TDW treatments exhibited lower final biomass concentrations, reaching 0.66 ± 0.10 g L⁻^1^ (TDW1), 0.55 ± 0.10 g L⁻^1^ (TDW2), and 0.68 ± 0.04 g L⁻^1^ (TDW3). However, biomass productivity was highest in TDW1 (42.54 ± 8.50 mg L⁻^1^ d⁻^1^), exceeding that observed in RDW treatments. Likewise, the highespecific growth rate was recorded in TDW1 (0.16 ± 0.01 d⁻^1^), followed by TDW2 (0.13 ± 0.01 d⁻^1^) and TDW3 (0.11 ± 0.01 d⁻^1^), indicating faster biomass turnover under lower initial inoculum densities in TDW.

The results indicate that the initial inoculum density may have influenced growth dynamics, biomass productivity, final biomass concentration, and nutrient removal, although the response was not uniform across the wastewater conditions evaluated. In the case of RDW, increasing the initial inoculum density from 10 to 30% resulted in a modest increase in final biomass concentration, rising from 0.73 to 0.78 g L⁻^1^. Conversely, the lower inoculum density in TDW resulted in the highest specific growth rate and biomass productivity (TDW1: *μ* = 0.16 d⁻^1^ and 42.54 mg L⁻^1^ d⁻^1^), whereas the higher inoculum density exhibited a lower specific growth rate (TDW3: *μ* = 0.11 d⁻^1^). This pattern is consistent with the influence of initial cell density on light availability within the culture. High cell concentrations can increase light attenuation and reduce the light available to individual cells, potentially affecting growth dynamics and productivity (Bohutskyi et al. 2016; Martínez et al. 2018). Furthermore, the composition and activity of the associated microbial community may vary according to microalgal density and wastewater conditions, potentially contributing to differences in overall system performance (Petrini et al. 2020; Yang et al. 2023).

The effect of inoculum density was also evident in phosphate removal from TDW, ranging from 98.56% reduction at the lowest inoculum density (TDW1) to 7.59% at the highest (TDW3). This result is consistent with the dependence of phosphorus removal on culture growth dynamics and cellular nutrient uptake, as reported for microalgal systems (Ale et al. 2014; Latiffi et al. 2017; Lau et al. 1995). However, since the present study did not directly quantify intracellular phosphorus accumulation, photosynthetic activity, or light distribution within the cultures, it is not possible to quantitatively distinguish the mechanisms underlying this density-dependent response. Consistent with these observations, the highest biomass densities in the nutrient-rich but turbid primary (untreated) effluent and the highest growth rates in the less turbid secondary (pre-treated) effluent at the lowest inoculum dose have been reported evaluating inoculum size and light intensity in primary and secondary municipal wastewater with *Chlorella* and *Scenedesmus* (Bohutskyi et al. 2016). These results have been attributed to the lower growth rates in the untreated effluent due to its higher optical density and reduced light penetration, the same trade-off observed here between RDW (higher final biomass) and TDW1 (lowest inoculum, highest specific growth rate). A sufficiently high inoculum improved the ability of microalgae to compete with wastewater-borne bacteria for nutrients and organic carbon, which is consistent with the inoculum-dependent performance of the present non-sterile system (Bohutskyi et al. 2016). Despite some studies reporting higher volumetric productivity at higher inoculum doses (Bohutskyi et al. 2016), the highest productivity here occurred at the lowest inoculum in TDW; this divergence may reflect differences in effluent nutrient status, microalgal species, and cultivation scale and duration.

In summary, the results demonstrate that the initial inoculum density had distinct effects on the evaluated process variables. A lower inoculum density was associated with higher specific growth rates, biomass productivity, and phosphate removal under the evaluated TDW condition, whereas a higher inoculum density resulted in a slightly higher final biomass concentration in RDW. Thus, the effect of inoculum density depended on the wastewater condition and the performance criterion considered, rather than following a uniform response across all variables. In this study, the native *Tetradesmus obliquus* strain demonstrated the ability to recover nutrients from both RDW and TDW while simultaneously producing substantial biomass (> 550 mg L⁻^1^). This performance highlights its suitability for integration into circular bioeconomy strategies, particularly in systems aiming to balance wastewater treatment efficiency with biomass generation under cost-effective operational conditions.

### Differences in microalgal biomass composition

**Fig. 7:**
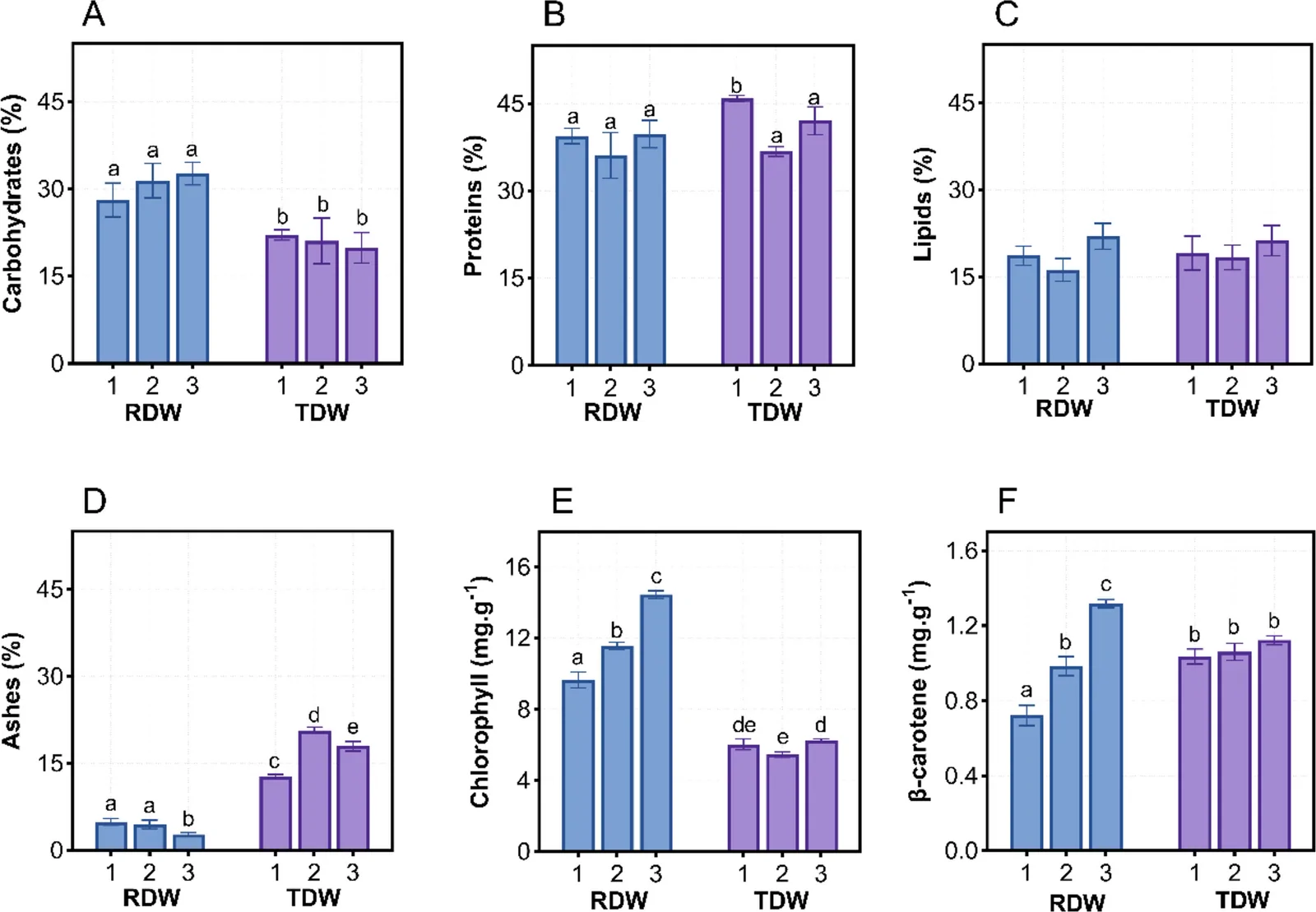
Biochemical composition of *Tetradesmus obliquus* dry biomass cultivated at three initial inoculum densities (1: 10%; 2: 20%; 3: 30%) in different types of domestic wastewater (RDW: raw domestic wastewater; TDW: primary-treated domestic wastewater). **A**. Total carbohydrate content. **B**. Total protein content. **C**. Total lipid content. **D**. Total ash content. **E**. Total chlorophyll concentration.** F**. *β*-Carotene concentration. Different letters indicate statistically significant differences among treatments (*p* < 0.05). Data are presented as mean ± standard deviation (*n* = 3)

The biochemical composition of *Tetradesmus obliquus* biomass cultivated in RDW and TDW under different initial inoculum densities is shown in Fig. 7. Overall, biomass composition differed primarily as a function of wastewater type, with initial inoculum density exerting a secondary but measurable influence.

Carbohydrate content (Fig. 7A) ranged from 19.93% to 32.67%, with significantly higher values observed in RDW compared with TDW (*p* < 0.001). This pattern is likely associated with the higher organic carbon and nutrient load in RDW, which may promote mixotrophic metabolism and favor carbohydrate synthesis and storage, consistent with reports describing enhanced carbon accumulation under mixotrophic conditions (Kamalanathan et al. 2018). Protein content (Fig. 7B) varied between 36.09% and 45.83%, with significantly higher values observed in TDW1 (p = 0.001). Similar protein levels have been reported for *Scenedesmus quadricauda* cultivated under mixotrophic conditions with white light-emitting diode (LED) illumination, where protein and carbohydrate contents of 45.18% and 23.03%, respectively, were obtained (Korozi et al. 2023). Total lipid content (Fig. 7C) ranged from 16.20% to 22.03%, with no statistically significant differences among treatments. The highest lipid fractions were observed in RDW3 (22.03%) and TDW3 (21.27%). These values are comparable to those reported in the literature from 13.90–15.20% (Tan et al. 2024) and 16.98% (Korozi et al. 2023), or lower to those reported in the literature at 30.70% (Pandey et al. 2019) for members of the *Scenedesmaceae* family. Thus, under the conditions evaluated, the biomass exhibited a composition characterized by relatively higher carbohydrate and protein contents than lipid contents. Ash content (Fig. 7D) ranged from 2.77% to 20.71%, with significantly higher values observed in TDW treatments (*p* < 0.0001). The higher ash content indicates differences in the inorganic fraction of the biomass between wastewater conditions. However, because the contribution of individual elements to the ash fraction was not quantified, the mechanisms underlying this difference cannot be resolved from the present data (P.S et al. 2021).

Total chlorophyll content (Fig. 7E) varied from 6.25 to 14.46 mg g⁻^1^, with significantly higher concentrations detected in RDW (*p* < 0.0001). These values are consistent with previous studies and may be associated with higher nutrient availability and increased optical density in RDW, both of which can stimulate chlorophyll synthesis (Ferreira et al. 2016; Mastropetros et al. 2022). In addition, reduced light availability has been reported to promote pigment accumulation as a photoacclimation response (Ferreira et al. 2016). *β*-carotene content (Fig. 7F) ranged from 0.72 to 1.32 mg g⁻^1^, with inoculum density accounting for most of the observed variance (*p* < 0.0001). The highest concentrations were observed in RDW3, followed by TDW3, consistent with reports indicating that carotenoid accumulation is often enhanced under shading or stress-related conditions that improve photoprotective capacity (Novoveská et al. 2019; Cheng et al. 2021). The values obtained are within the range reported in the literature, where concentrations up to 2 mg g⁻^1^ have been described (Ren et al. 2021).

The contrasting biochemical profiles of the biomass produced in RDW and TDW indicate that the two wastewater matrices were associated with distinct patterns of biomass composition. Biomass cultivated in RDW, which exhibited higher turbidity (OD₆₈₀ = 0.34) and lower specific growth rates (µ = 0.09–0.10 d⁻^1^), contained significantly higher carbohydrate concentrations, reaching 32.68% (*p* < 0.001). This pattern is consistent with differences in carbon allocation under contrasting cultivation conditions and with the accumulation of storage carbohydrates reported for *Scenedesmus* and *Tetradesmus* under conditions of organic carbon availability (Kamalanathan et al. 2018; Sun et al. 2018; Cheng et al. 2021). The higher chlorophyll content observed in RDW biomass, reaching 14.46 mg g⁻^1^ (*p* < 0.0001), may be related to the low light availability associated with high turbidity of this wastewater. In green microalgae, reduced irradiance can induce photoacclimation changes in the photosynthetic apparatus, including increased pigment levels and modifications to the organization of light-harvesting antennae, thereby enhancing the capacity to capture available photons under light-limiting conditions (Ferreira et al. 2016; Novoveská et al. 2019; Aizpuru and González-Sánchez 2024). This interpretation aligns with the higher chlorophyll content observed in RDW despite the lower specific growth rates; however, since the intracellular light field, photosynthetic activity, and antenna composition were not measured, the occurrence of photoacclimation cannot be directly demonstrated in the present study. The higher *β*-carotene content detected in RDW may also reflect differences in pigment regulation associated with the distinct physiological conditions of the cultures. However, the carotenoid response to light availability depends on the specific pigment and conditions, and current data do not allow for the determination of the specific mechanism responsible for the higher *β*-carotene content. In *Scenedesmus*, chlorophyll and carotenoid metabolism are dynamically regulated according to physiological status and culture conditions, with carotenoids participating in both the photosynthetic apparatus and photoprotection (Maroneze et al. 2019).

The detection of iron (0.69 mg L⁻^1^) and manganese (0.14 mg L⁻^1^) exclusively in RDW provides additional physiological context for the observed differences in pigments. Iron is required for various components of the photosynthetic electron transfer system and is associated with chlorophyll and carotenoid metabolism, whereas manganese is an essential component of the oxygen-evolving complex of photosystem II (PSII) (MacIntyre et al. 2002; Aizpuru and González-Sánchez 2024). Consequently, the presence of these elements in RDW may have contributed to the maintenance of the photosynthetic apparatus. However, the contribution of iron and manganese to pigment accumulation cannot be established based on current data, as intracellular metal content, metal bioavailability, pigment biosynthesis fluxes, and photosynthetic performance were not measured. Therefore, the higher pigment content in RDW should be interpreted as the result of a response consistent with differences in light availability and nutrient composition, rather than as evidence of a demonstrated causal mechanism.

In TDW, the reciprocal pattern, higher protein and a fatty-acid pool dominated by polyunsaturated rather than saturated and monounsaturated acids, is consistent with a more light-replete, faster-growing physiology. The protein maximum was specific to TDW1 (up to 45.83%, p = 0.001), the treatment with the highest specific growth rate (µ = 0.16 d⁻^1^) and the lowest inoculum density. Under these conditions, carbon and assimilated nitrogen may be preferentially co-routed toward protein synthesis rather than toward storage carbohydrate. This partitioning may involve the GS–GOGAT cycle, the primary pathway for ammonium assimilation in microalgae. In this cycle, glutamine synthetase incorporates ammonium into glutamate to form glutamine in an ATP-dependent reaction, whereas glutamate synthase transfers the amide group from glutamine to 2-oxoglutarate, generating glutamate and coupling nitrogen assimilation with carbon skeletons derived from central metabolism. Under conditions supporting rapid cell proliferation and adequate nutrient availability, increased synthesis of nitrogen-rich cellular components, including ribosomal proteins and photosynthetic proteins such as Rubisco, may contribute to higher cellular protein content (Mastropetros et al. 2022; Korozi et al. 2023). This interpretation applies specifically to TDW1 and should be made with caution for TDW2 and TDW3, where the pH exceeded 10 (Fig. 3A); at such pH, ammonia volatilization and a shift of inorganic-carbon speciation toward carbonate may limit nitrogen and carbon availability, so the higher-pH treatments are not directly comparable.

As to the lipid fraction, TDW biomass showed a higher relative proportion of C18 polyunsaturated fatty acids, chiefly α-linolenic (C18:3) and linoleic (C18:2) acids, whereas RDW biomass was dominated by palmitic (C16:0) and oleic (C18:1) acids. Because the total lipid content did not differ significantly between treatments, the observed differences represent an alteration in the relative composition of the fatty acid pool rather than a proven increase in the absolute PUFA content. In microalgae, C18 polyunsaturated fatty acids are characteristic components of the thylakoid galactolipids monogalactosyldiacylglycerol and digalactosyldiacylglycerol, whereas triacylglycerols constitute the primary lipid storage compounds (Li-Beisson et al. 2019). In this context, the higher relative abundance of PUFAs in TDW biomass may reflect a greater contribution of photosynthetic membrane lipids under conditions associated with higher light availability and faster growth. Conversely, the higher relative abundance of saturated and monounsaturated fatty acids in RDW biomass may be consistent with a greater contribution of storage-associated lipids, given the carbon-rich conditions of this raw wastewater matrix (Piasecka et al. 2020; Tan et al. 2024). However, since lipid classes were not directly quantified, this proposed relationship remains an interpretation of the observed fatty acid profiles rather than a proven alteration in membrane or storage lipid composition.

Overall, these results are consistent with previous findings indicating that increased nutrient availability favors pigment and protein synthesis, whereas elevated organic carbon availability promotes carbohydrate accumulation (Kamalanathan et al. 2018; Novoveská et al. 2019; Mastropetros et al. 2022; Tan et al. 2024). The biochemical divergence observed between RDW and TDW grown biomass highlights the adaptive capacity of the native *Tetradesmus obliquus* strain, likely driven by differential regulation of photosynthetic and stress-response pathways modulated by effluent composition and inoculum density (Sun et al. 2018; Singh et al. 2024). From an application perspective, RDW favored the production of biomass enriched in carbohydrates and pigments, whereas TDW promoted higher protein content and mineral accumulation, suggesting distinct valorization routes for the biomass depending on the wastewater type (Patnaik et al. 2019; Braun and Colla 2023).

### Fatty acid composition

The fatty acid profiles of *Tetradesmus obliquus* biomass cultivated in RDW and TDW are summarized in Table 3 and reveal clear differences between the two wastewater types, indicating distinct lipid partitioning patterns. Among saturated fatty acids (SFAs), palmitic acid (C16:0) was predominant, with the highest proportions observed in RDW2 (35.35%) and RDW3 (32.74%), followed by myristic acid (C14:0), which was particularly abundant in RDW2 (8.07%). These SFAs are commonly reported as major components of microalgal lipids and are typically associated with biomass grown under carbon-rich conditions. Monounsaturated fatty acids (MUFAs) were mainly represented by oleic acid (C18:1), which predominated in RDW1 (29.59%) and RDW2 (21.54%). In contrast, TDW-grown cultures exhibited a high relative abundance of polyunsaturated fatty acids (PUFAs), particularly linoleic acid (C18:2; mean 15.21%) and α-linolenic acid (C18:3; mean 37.30%). This shift toward more unsaturated lipid fractions is consistent with previous observations indicating that microalgae cultivated under variable or nutrient-modulated conditions tend to increase fatty acid desaturation as part of their physiological acclimation response. Notably, relatively high proportions of long-chain PUFAs were detected in the TDW treatments, including putative docosahexaenoic acid (DHA, C22:6; up to 10.68%) and docosapentaenoic acid (DPA, C22:5; up to 11.23%). Although these fatty acids are not commonly reported as major constituents of *Tetradesmus* biomass, long-chain PUFA signals have been sporadically described under specific cultivation conditions. The relative proportions observed here were higher than those reported in previous studies under optimal growth conditions (Pandey et al. 2019; El-Sheekh et al. 2023) and in salt-stress experiments (Pandit et al. 2017), suggesting that wastewater-based cultivation may influence elongation and desaturation pathways involved in PUFA biosynthesis. Nevertheless, further targeted studies would be required to elucidate the metabolic mechanisms underlying the accumulation of long-chain PUFAs in this genus.

**Table 3:** Relative fatty-acid composition of *Tetradesmus obliquus* biomass cultivated at three initial inoculum densities in raw domestic wastewater (RDW) and primary-treated domestic wastewater (TDW)

Fatty acid		Area (%)					
	n°C:nº	TDW1	TDW2	TDW3	RDW1	RDW2	RDW3
Myristic acid	C14:0	0.66	0.30	0.67	1.74	8.07	2.14
Docosapentaenoic acid (DPA)	C22:5	–	–	11.23	4.66	–	5.96
Linoleic acid	C18:2	16.92	14.49	15.21	20.71	13.98	1.11
Palmitelaidic acid	C16:1	0.42	1.47	–	6.65	2.73	6.89
Palmitic acid	C16:0	18.24	13.49	16.78	22.20	35.35	32.74
Octadecatetraenoic acid	C18:4	4.75	4.34	3.97	1.18	0.86	1.45
Oleic acid	C18:1	–	3.35	3.12	34.97	26.97	–
8,11-octadecadienoic acid	C18:2	–	–	–	1.69	1.09	4.41
Stearic acid	C18:0	1.44	2.31	2.45	4.06	2.66	2.29
Pentadecanoic acid	C15:0	0.38	0.17	0.27	–	–	–
Docosahexaenoic acid (DHA)	C22:6	10.26	10.68	–	–	3.40	–
Palmitoleic acid	C16:1	1.63	–	1.27	–	–	1.23
Hexadecadienoic acid	C16:2	1.02	0.87	–	–	–	1.45
Hexadecatrienoic acid	C16:3	–	8.39	7.35	–	–	–
Linolenic acid	C18:3	2.11	1.93	1.64	–	–	1.50
Heptadecatrienoic acid	C17:3	-	–	–	–	–	5.70
α-linolenic acid	C18:3	37.66	38.21	36.04	–	–	–
Cis-6-octadecenoic acid	C18:1	–	–	–	0.97	–	–
Stearolaidic acid	C18:1	–	–	–	1.17	–	–
Elaidic acid	C18:1	4.51	–	–	–	1.28	25.82
Trans-8-octadecenoic acid	C18:1	-	–	-	–	2.57	3.61
Eicosadienoic acid	C20:2	–	–	–	–	1.04	–
10-ketostearic acid	C18:0	-	–	–	–	–	3.70
∑SFA*		20.72	16.27	20.17	28.00	46.08	40.87
∑MFA**		6.56	4.82	4.39	43.76	33.55	37.64
∑PUFA***		72.72	78.91	75.44	28.24	20.37	21.58

*∑SFA, sum of saturated fatty acids; *∑MUFA, sum of monounsaturated fatty acids; *∑PUFA, sum of polyunsaturated fatty acids

In this study, TDW grown cultures consistently accumulated more PUFAs, aligning with other observations in the literature: PUFA levels of 42.60% in *Scenedesmus obliquus* cultivated in wastewater (Tan et al. 2024) and *Tetradesmus obliquus* under phosphate limitation can accumulate up to 46.65% of PUFAs (Roy et al. 2021). A fatty-acid profile in which palmitic, linoleic and linolenic acids together accounted for about 75% of total methyl esters, analyzing *Chlorella sorokiniana* grown in wastewater, without detectable long-chain (C20–C22) PUFAs (Bohutskyi et al. 2016), a comparatively simple, C16/C18-based profile relative to the higher relative proportion of C18 polyunsaturated fatty acids observed in the TDW biomass of the present study.

Beyond the specific results of this study, microalgal biomass supports a diverse portfolio of real-world applications. Protein-rich fractions are used as ingredients in aquaculture and animal feeds, with *Scenedesmus*/*Tetradesmus* biomass having been evaluated as a partial substitute for fishmeal (Patnaik et al. 2019); PUFA-rich oils are of interest as nutraceutical sources of omega-3 (Nigam et al. 2018; Piasecka et al. 2020); and the biomass is also utilized to produce biostimulants, biofertilizers, natural pigments, and bioenergy feedstocks, while (when cultivated in wastewater) enabling the simultaneous recovery of nutrients, effluent treatment, and CO₂ capture (Levasseur et al. 2020; Gouveia et al. 2016). The main advantages of this platform are the lack of competition for arable land, rapid photoautotrophic growth, and the ability to integrate effluent bioremediation with biomass valorization. These benefits are balanced by recognized limitations: the energy consumption and costs associated with harvesting and dewatering dilute cultures, light limitation in turbid or colored media, the risk of contamination in non-axenic systems, seasonal and operational variability, and downstream processing costs (Acién Fernández et al. 2019; Novoveská et al. 2023; Borowitzka and Vonshak 2017; Gouveia et al. 2016).

The fatty acid profiles observed in this study suggest that the cultivation matrix may influence the potential valorization pathways of the resulting biomass. Raw domestic wastewater biomass was characterized by higher relative proportions of palmitic acid (C16:0, up to 35.35%) and oleic acid (C18:1, up to 34.97%), whereas TDW biomass showed a greater relative contribution of the C18 polyunsaturated fatty acids α-linolenic acid (C18:3, approximately 36–38%) and linoleic acid (C18:2, approximately 15%). Palmitic and stearic acids are relevant to biodiesel production because of their contribution to oxidative stability, whereas oleic acid is generally associated with a favorable balance between oxidative stability and low-temperature properties. Linoleic and α-linolenic acids are essential polyunsaturated fatty acids with recognized nutritional value and potential applications in aquafeeds and nutraceutical products (Nigam et al. 2018; Piasecka et al. 2020). However, the fatty acid data obtained here represent relative peak-area percentages rather than absolute yields; therefore, these results indicate compositional differences among treatments but do not establish differences in the absolute production of individual fatty acids. From a circular-bioeconomy perspective, the use of domestic wastewater as a nutrient source may reduce the need for externally supplied nutrients and trace elements, potentially lowering the resource requirements and production costs of microalgal cultivation (Acién Fernández et al. 2019). Thus, the simultaneous recovery of nutrients from wastewater and production of biomass with distinct biochemical profiles supports the potential integration of microalgal bioremediation with biomass valorization strategies, although the economic feasibility and suitability of specific downstream applications require further process-specific evaluation (Patnaik et al. 2019; Levasseur et al. 2020).

Despite the nutrient removal achieved and the production of protein- and PUFA-rich biomass, several limitations constrain the large-scale application of microalgae-based domestic wastewater treatment. Effluent variability, turbidity-related light limitation, non-sterile cultivation, and the energy and cost requirements associated with biomass harvesting and downstream processing remain important challenges. In addition, the potential accumulation of trace metals and other contaminants may restrict the end uses of wastewater-grown biomass and requires application-specific assessment. Recent analyses place wastewater-based microalgal production at a demonstration-to-pilot stage, identifying dilute biomass harvesting and energy consumption as major economic bottlenecks, with production costs varying widely according to effluent composition and productivity (Dias et al. 2025). Future research should therefore prioritize pilot-scale validation under variable operating conditions, low-cost harvesting strategies, microbiome and strain-stability monitoring, and integrated techno-economic and life-cycle assessments. The integration of wastewater treatment with cascade biorefinery approaches may improve process viability by recovering multiple biomass-derived products, although this potential remains to be validated under realistic operating conditions.

## Conclusions

This study isolated and molecularly identified a native strain of *Tetradesmus obliquus* from domestic wastewater in southern Brazil. The level of effluent treatment and the initial inoculum density were associated with differences in nutrient and trace metal removal, biomass productivity, and biochemical composition. The strain grew in both RDW and TDW; biomass produced in raw effluent exhibited higher carbohydrate and pigment contents, whereas primary-treated effluent yielded biomass with higher protein content and a greater relative proportion of PUFAs. Since fatty acids were characterized based on relative peak areas in gas chromatography–mass spectrometry (without the use of authentic standards), the results indicate differences in fatty acid composition rather than confirmed differences in absolute yields; furthermore, the identification of atypical long-chain PUFAs remains preliminary. Overall, the results support the use of domestic wastewater as a nutrient source for microalgae cultivation, simultaneously enabling nutrient recovery and the production of biomass rich in proteins and PUFAs. Further pilot-scale studies are required, considering seasonal variability, cultivation stability, and techno-economic and life-cycle assessments, to evaluate the feasibility of scaling up the process.

## Data Availability

The data supporting the findings of this study are available within the article.
